# Yeast Efficiently Utilizes Ribosomal RNA-Derived Oligonucleotides as Bioavailable Nutrient Sources

**DOI:** 10.3390/foods15020318

**Published:** 2026-01-15

**Authors:** Xinmei Du, Qitao Chen, Jingyun Zhuang, Mingqi Zhao, Yixin Duan, Shuang Wang, Ran An, Xingguo Liang

**Affiliations:** 1 College of Food Science and Engineering, Ocean University of China, Qingdao 266404, China; duxinmei@stu.ouc.edu.cn (X.D.); chenqitao2393@stu.ouc.edu.cn (Q.C.); zhuangjingyun@stu.ouc.edu.cn (J.Z.); zhaomingqi@stu.ouc.edu.cn (M.Z.); duanyixinn@163.com (Y.D.); shuang.wang@ouc.edu.cn (S.W.); 2 Laboratory for Marine Drugs and Bioproducts, Qingdao Marine Science and Technology Center, Qingdao 266237, China

**Keywords:** yeast, rRNA-derived oligonucleotides, oligonucleotide nutrient sources, endocytosis, autophagy, proteomics

## Abstract

Nucleic acids are essential dietary components with diverse physiological functions. Numerous studies have focused on the biological functions of nucleotides, nucleosides, and functional RNAs such as microRNAs. However, the nutritional value of ribosomal RNA (rRNA)-derived oligonucleotides, which are likely the predominant nucleic acid-derived components in foods, remains largely unexplored. Here, yeast was used as a food-associated eukaryotic model organism to investigate the uptake and utilization of rRNA-derived oligonucleotides. Yeast efficiently utilized short RNA oligonucleotides (approximately 5–30 nt) as nutrient sources, supporting robust cell growth. Confocal microscopy confirmed rapid uptake of Cy5-labeled RNA oligonucleotides by yeast cells. Proteomic analysis further revealed marked upregulation of proteins involved in endocytosis and autophagy in yeast cultured with RNA oligonucleotides. Collectively, these findings demonstrate that yeast can internalize and metabolize rRNA-derived oligonucleotides as efficient nutrient sources, likely through coordinated endocytic and autophagic pathways. This study highlights the nutritional potential of rRNA-derived oligonucleotides and provides a foundation for their future application in functional foods and fermentation systems.

## 1. Introduction

Nucleic acids are abundant in a wide range of foods, including meat, fish, and vegetables, and play diverse physiological and biochemical roles [1,2,3]. In particular, nucleotides and nucleosides have been reported to exert multiple biological functions, such as promoting intestinal epithelial cell differentiation [4,5], supporting cellular energy metabolism [6,7,8], and modulating gut microbiota composition [2,9]. In the food industry, nucleotides are widely applied as flavor enhancers [10], ingredients in infant formula [11,12], and components of foods for special medical purposes (FSMPs) [13]. With the growing applications of nucleic acids in food systems, the nutritional value of dietary nucleic acids has attracted increasing attention.

Recent studies have revealed that certain dietary microRNAs can be directly absorbed by mammalian cells through specialized transport pathways, such as SIDT1-mediated uptake and exosomal delivery [14]. Once internalized, these microRNAs can regulate gene expression across species [15,16,17]. However, microRNAs account for only a minor proportion of total cellular RNA (0.003–0.02%) [18], while ribosomal RNA (rRNA) constitutes nearly 80% [19]. Despite the high abundance of rRNA in foods and biological materials, most previous studies have focused on its biological functions rather than its nutritional implications.

Moreover, RNA is susceptible to enzymatic degradation and to non-enzymatic cleavage induced by heat, pH fluctuations, and mechanical stress during food processing, cooking, and storage [20,21,22]. Consequently, rRNA in foods is readily fragmented into diverse oligonucleotides. These rRNA-derived oligonucleotides may therefore represent the predominant form of dietary RNA entering the digestive system. Moreover, they are likely accessible to eukaryotic microorganisms in food and fermentation systems, such as yeasts. Despite their widespread occurrence, it remains unclear whether such RNA oligonucleotides can function as utilizable nutrients for eukaryotic cells, an essential question for understanding the nutritional significance of dietary nucleic acids.

In this study, *Komagataella phaffii* X-33 (*K. phaffii* X-33), a food-associated eukaryotic model organism and widely used industrial fermentation strain [23,24,25], was used to investigate whether rRNA-derived oligonucleotides can serve as nutritional components and how they are taken up and metabolized at the cellular level. Our findings demonstrate that short RNA oligonucleotides can be efficiently utilized by yeast as nutrient sources, offering new insights into nucleic acid nutrition and its potential applications in functional foods and fermentation systems.

## 2. Materials and Methods

### 2.1. Materials

#### 2.1.1. Yeast Strain and Culture Media Composition

The yeast strain *K. phaffii* X-33 was obtained from the China General Microbiological Culture Collection Center (CGMCC, Beijing, China). Culture medium components, including glucose, peptone, yeast extract, K_2_HPO_4_, MgSO_4_, NaCl, and NaNO_3_, as well as the constituents of 0.2 M phosphate-buffered saline (PBS; 0.2 M Na_2_HPO_4_·12H_2_O and 0.2 M NaH_2_PO_4_·2H_2_O, pH 7.4), were purchased from China National Pharmaceutical Group Co., Ltd. (Shanghai, China).

#### 2.1.2. Nucleic Acid Materials

Nucleotides (NT) and nucleosides (NS), derived from RNA hydrolysis of *Saccharomyces cerevisiae* (*S. cerevisiae*), were purchased from Nantong QZU Bioscience & Biotechnology Co., Ltd. (Nantong, China).

Two RNA oligonucleotide preparations derived from *S. cerevisiae* rRNA hydrolysis were used. Mix-A contained both RNA oligonucleotides (<30 nt; 20–35%) and nucleotides (65–80%) and was supplied by Nanjing Tongkai Zhaoye Biotechnology Co., Ltd. (Nanjing, China). Mix-B was a high-purity RNA oligonucleotide preparation (>90% RNA oligonucleotides 10–100 nt and <1% nucleotides) purchased from Beijing Solabio Technology Co., Ltd. (Beijing, China).

Chemically synthesized RNAs (8, 12, and 20 nt) and Cy5-labeled 20 nt RNA were synthesized by Aiji Biotechnology Co., Ltd. (Guangzhou, China), with sequences based on the *S. cerevisiae* 18S rRNA region (Table S4).

#### 2.1.3. Chemical Reagents

RNase A and RNase inhibitor were purchased from Beijing Solabio Technology Co., Ltd. (Beijing, China) and ABclonal (Wuhan, China), respectively. RNA ladder (10–50 nt) was obtained from Biyuntian Biotechnology Co., Ltd. (Shanghai, China). TRIzol reagent (Thermo Fisher Scientific, Waltham, MA, USA), FastKing RT Kit (TianGen, Beijing, China), and BlasTaq 2× qPCR Master Mix (ABM, Richmond, BC, Canada) were used for RNA extraction and RT-qPCR. Calcofluor White (CFW) was purchased from Shanghai Yuanye Biotechnology Co., Ltd. (Shanghai, China), and 4% paraformaldehyde from Lanjieke Technology Co., Ltd. (Beijing, China). SDS, tris(2-carboxyethyl)phosphine (TCEP), iodoacetamide (IAA), acetonitrile, and formic acid were obtained from Fisher Scientific (Waltham, MA, USA). The BCA Protein Assay Kit was purchased from Thermo Fisher Scientific (Waltham, MA, USA). Hydrophilic–lipophilic balance (HLB) cartridges were obtained from Waters (Milford, MA, USA), and sequencing-grade trypsin (enzyme activity ≥ 250 units/mg) from Promega (Madison, WI, USA).

### 2.2. Yeast Cultivation

#### 2.2.1. Yeast Seed Culture Preparation

Yeast was initially streaked onto YPD solid medium (20 g/L glucose, 20 g/L peptone, 10 g/L yeast extract, and 20 g/L agar) and incubated at 28 °C under aerobic conditions for 3 days. Single colonies were transferred to liquid basal medium (0.5 g/L K_2_HPO_4_, 0.5 g/L MgSO_4_, 0.5 g/L NaCl, 5 g/L or 20 g/L glucose, and 1 g/L NaNO_3_) and incubated at 28 °C with shaking at 150 rpm for 3 days. After incubation, cells were harvested by centrifugation (10,000 rpm, 4 °C, 5 min) and washed three times with 0.2 M PBS to remove residual glucose and NaNO_3_. The washed cells were resuspended in the same buffer to obtain the yeast seed culture (OD_600_ ≈ 0.5).

#### 2.2.2. Yeast Culture with Nucleic Acid Sources

For media containing nucleic acids, including RNA oligonucleotides (Mix-A or Mix-B), nucleotides, and nucleosides, components were sterilized by filtration (0.22 µm) and added to carbon- and/or nitrogen-free basal medium at final concentrations of 1–45 g/L. The yeast seed culture was inoculated into 60 mL of the medium at an inoculum volume of 5% (*v*/*v*) and incubated at 28 °C under aerobic conditions with shaking at 150 rpm. Yeast growth was monitored by OD_600_ using a Varioskan Flash microplate reader (Thermo Scientific, Waltham, MA, USA), and viable cell numbers were determined using the plate count method. Growth rate (divisions per day) was calculated as [(lg X_2_ − lg X_1_)/lg 2]/(t_2_ − t_1_), where X_1_ and X_2_ represent yeast cell counts at times t_1_ and t_2_, respectively.

#### 2.2.3. Yeast Culture with Chemically Synthesized RNA as the Sole Nitrogen Source

When chemically synthesized RNA was used as the sole nitrogen source, 0.8 g/L of RNA (8 nt, 12 nt, or 20 nt) was added separately to 200 µL of basal medium. RNase inhibitor (0.3 U/µL) was added to prevent RNA degradation. Yeast seed culture was inoculated at 5% (*v*/*v*) and incubated at 28 °C under aerobic conditions with shaking at 150 rpm. Yeast cell numbers were determined using a hemocytometer.

### 2.3. Quantification of Nucleic Acid Substrates in Culture Supernatants (OD_260_)

Yeast cells were cultured in basal medium containing nucleic acid substrates (Mix-A, Mix-B, nucleotides, and nucleosides). At designated time intervals, cultures were centrifuged (10,000 rpm, 4 °C, 5 min) to collect cell-free supernatants. The concentration of residual nucleic acid substrates in the supernatant was measured by absorbance at 260 nm (OD_260_) using a NanoDrop 2000 spectrophotometer (Thermo Scientific, Waltham, MA, USA).

### 2.4. RNA Oligonucleotide Length Distribution Analysis

#### 2.4.1. Denaturing Polyacrylamide Gel Electrophoresis (dPAGE)

Denaturing polyacrylamide gel electrophoresis (15% dPAGE, 8 M urea) was used to determine RNA oligonucleotide length distribution. Mix-A (33.3 g/L) and Mix-B (0.6 g/L) samples were loaded at 4 µL per lane. RNA bands were visualized and quantified using Image Lab software (version 3.0 build 11, Bio-Rad Laboratories, Hercules, CA, USA), with an RNA ladder (10–50 nt) as the molecular size marker.

#### 2.4.2. Capillary Electrophoresis (CE)

Mix-A or Mix-B was diluted to 2 ng/μL with RNase-free water, and 20 μL of each sample was analyzed using a Qsep100 capillary electrophoresis system (BiOptic Inc., New Taipei City, Taiwan, China) equipped with an S2 standard cartridge. A 1.0 μL aliquot of each sample was injected at 4 kV for 10 s, and separation was performed at 8 kV for 200 s in Tris-boric acid-EDTA buffer.

### 2.5. HPLC Analysis of RNA Oligonucleotide Composition

Mix-A and Mix-B were analyzed using a YMC-Pack ODS-AQ C18 column (4.6 mm × 250 mm) on a Hitachi Chromaster HPLC system (Hitachi High-Technologies, Tokyo, Japan). Mobile phase A consisted of 50 mM ammonium formate (pH 6.45), and mobile phase B was 50% acetonitrile containing 50 mM ammonium formate (pH 6.45). The gradient elution program was as follows: 0–8 min, 10% B; 8–9 min, 10–12% B; 9–19 min, 12% B; 19–24 min, 12–20% B; 24–29 min, 20–100% B; 29–39 min, 100% B; 39–40 min, 100–10% B; 40–70 min, 10% B. The flow rate was 0.5 mL/min, and the column temperature was maintained at 25 °C. Detection was performed at 260 nm.

### 2.6. Enzymatic Digestion of RNA Oligonucleotides by RNase A

Mix-B (10 g/L or 50 g/L) was incubated with RNase A (0.1 g/L or 0.5 g/L) at 28 °C for 3 days in a total volume of 20 mL. Digestion products were analyzed by denaturing polyacrylamide gel electrophoresis (15% PAGE, 8 M urea), and band intensities were quantified using Image Lab software (version 3.0 build 11, Bio-Rad, Hercules, CA, USA). An RNA ladder (10–50 nt) was used as a molecular size marker.

### 2.7. RNA Extraction and RT-qPCR

Yeast cells were cultured for 4 and 6 days under the conditions described above prior to RNA extraction. A 1 mL aliquot of culture (OD_600_ ≈ 1.0) was centrifuged (10,000 r/min, 4 °C, 5 min), and total RNA was extracted using TRIzol reagent (Thermo Fisher Scientific, Waltham, MA, USA) following the manufacturer’s instructions. RNA quality and concentration were determined by the NanoDrop 2000 spectrophotometer (Thermo Fisher Scientific, USA). Samples with an OD_260_/OD_280_ ratio between 1.9 and 2.0 were used for analysis.

For cDNA synthesis, 50 ng of total RNA was reverse-transcribed in a 10 μL reaction volume using the FastKing RT Kit (Tiangen Biotech, Beijing, China). RT-qPCR was performed on a PikoReal 96 Real-Time PCR System (Thermo Fisher Scientific, Waltham, MA, USA) with BlasTaq 2× qPCR Master Mix (ABM, Richmond, BC, Canada). Each 10 μL reaction contained 5 μL of 2× Master Mix, 1 μL of cDNA template, and 0.5 μM each forward and reverse primer.

The qPCR cycling conditions were as follows: initial denaturation at 94 °C for 3 min, followed by 40 cycles of 94 °C for 30 s, 60 °C for 30 s, and 72 °C for 45 s. *Glyceraldehyde-3-phosphate dehydrogenase* (*GAPDH*) served as the reference gene, and relative expression was calculated using the 2^−ΔCt^ method (ΔCt = Ct *_target gene_* − Ct *_GAPDH_*). Primer sequences are listed in Table S3.

### 2.8. Fluorescence Imaging of Cy5-Labeled RNA in Yeast

Yeast cells were cultured in basal medium (0.5 g/L K_2_HPO_4_, 0.5 g/L MgSO_4_, 0.5 g/L NaCl, and 20 g/L glucose) supplemented with 1 µM 5′-Cy5–RNA_(20)_ (5′-Cy5–AUCUCGACCCUUUGGAAGAG, 20 nt) and 0.3 U/µL RNase inhibitor. Cultures were incubated at 28 °C under aerobic conditions with vertical shaking (35 rpm).

After incubation, cells were washed three times with 0.2 M PBS, fixed with 4% paraformaldehyde (PFA) at room temperature for 15 min in the dark, and then stained with Calcofluor White (CFW) for 15 min. After washing, cells were resuspended in 0.2 M PBS, and 5 µL of cell suspension was mounted on a microscope slide with 2 µL of anti-fade reagent.

Fluorescence images were acquired using a Nikon A1R HD25 confocal laser scanning microscope (Nikon, Tokyo, Japan) equipped with a 60× oil-immersion objective (1024 × 1024 pixels). CFW fluorescence was excited at 402.7 nm (emission 430–480 nm), and Cy5 fluorescence was excited at 637.4 nm (emission 660–720 nm). Identical acquisition parameters were used for all samples. Image processing was performed using NIS-Elements Viewer (version 5.22.00 build 1485, Nikon, Tokyo, Japan), and fluorescence intensity was quantified using ImageJ software (version 1.54g, NIH, Bethesda, MD, USA).

### 2.9. Proteomic Analysis (4D-DIA)

#### 2.9.1. Protein Extraction

Yeast was cultured in basal medium containing Mix-A (5 g/L) or glutamate (5 g/L) as the sole nitrogen source. After 3 days, cells were harvested by centrifugation (10,000 rpm, 5 min, 4 °C), washed twice with 0.2 M PBS, and then frozen in liquid nitrogen. They were stored at −80 °C until use. Proteins were extracted using lysis buffer (8 M urea, 1% SDS, 50 mM Tris-HCl, pH 8.0) supplemented with protease inhibitors. Cell lysates were homogenized and centrifuged (12,000× *g*, 20 min, 4 °C), and supernatants were collected. Protein concentration was determined using the bicinchoninic acid (BCA) assay.

#### 2.9.2. Protein Digestion and Peptide Desalting

A total of 100 µg protein was reduced with 10 mM TCEP at 37 °C for 60 min, alkylated with 40 mM IAA in the dark at 25 °C for 40 min, and digested overnight with trypsin (enzyme-to-substrate ratio 1:50, *w*/*w*) at 37 °C. The resulting peptides were desalted using HLB cartridges, dried in a vacuum concentrator, and reconstituted in 0.1% formic acid for mass spectrometry analysis.

#### 2.9.3. DIA–MS Analysis

Peptides were separated on an EASY-nLC 1200 system (Thermo Fisher Scientific, Waltham, MA, USA) equipped with a C18 column (75 µm × 25 cm, Ionopticks, Fitzroy, VIC, Australia). Mobile phase A consisted of 2% acetonitrile and 0.1% formic acid, and mobile phase B contained 80% acetonitrile and 0.1% formic acid. The separation gradient was: 3–28% B over 0–33 min, 28–44% B over 33–37 min, 44–90% B over 37–40 min, followed by 90% B for 4 min. The flow rate was 250 nL/min.

Eluted peptides were analyzed on a timsTOF Pro2 mass spectrometer (Bruker Daltonics, Bremen, Germany) operated in positive ion mode. Data-independent acquisition (DIA) was performed with an ion source voltage of 1.5 kV and a mass range of *m*/*z* 100–1700. Accumulation and ramp times were both set to 100 ms, and the ion mobility coefficient (1/K_0_) ranged from 0.6 to 1.6 Vs·cm^−2^ using 64 DIA-PASEF windows.

#### 2.9.4. Bioinformatics Analysis

Raw DIA data were processed using Spectronaut software (version 14, Biognosys AG, Schlieren, Switzerland). Proteins were quantified based on up to six unique peptides per protein, with peptide abundance calculated as the sum of the peak areas of the three most intense fragment ions. Filtering parameters were set as Protein FDR ≤ 0.01, Peptide FDR ≤ 0.01, Peptide Confidence ≥ 99%, and XIC width ≤ 75 ppm. Proteins with *p* < 0.05 and fold change >2 or <0.5 were classified as differentially expressed proteins (DEPs). Proteomic data were uploaded to the Majorbio Cloud platform (https://cloud.majorbio.com) for KEGG functional annotation and KEGG pathway enrichment analysis.

### 2.10. Statistical Analysis

All experiments were performed in triplicate. Data are presented as mean ± standard deviation (SD). Statistical significance was evaluated using one-way analysis of variance (ANOVA) followed by the least significant difference (LSD) post hoc test (SPSS 25.0, IBM, Armonk, NY, USA). Differences were considered significant at *p* * < 0.05 and highly significant at *p* ** < 0.01. Values with *p* > 0.05 were considered not significant (n.s.). Graphs were generated using Origin software  (version 2023, OriginLab, Northampton, MA, USA), and electrophoresis band intensities were quantified using Image Lab software (version 3.0 build 11, Bio-Rad Laboratories, Hercules, CA, USA).

## 3. Results

### 3.1. Mixture of RNA Oligonucleotides and Nucleotides as Nitrogen Sources for Yeast Growth

**Figure 1:**
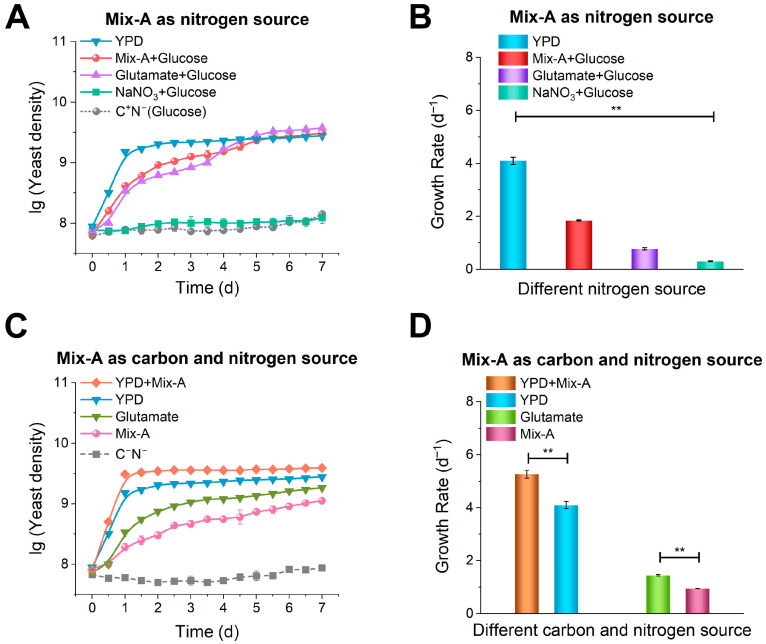
Yeast growth with Mix-A (oligonucleotide–nucleotide mixture) as the nitrogen source. (**A**) Yeast growth with Mix-A as the sole nitrogen source. (**B**,**D**) Growth rates under different media conditions. (**C**) Yeast growth occurred when Mix-A served as both the carbon and nitrogen sources. Media compositions: YPD (5 g/L yeast extract, peptone, and 5 g/L glucose); Mix-A + Glucose (5 g/L Mix-A and 5 g/L glucose); Glutamate + Glucose (5 g/L glutamate and 5 g/L glucose); NaNO_3_ + Glucose (5 g/L NaNO_3_ and 5 g/L glucose); C^+^N^−^ (glucose only, no nitrogen sources); YPD + Mix-A (5 g/L yeast extract and peptone, 5 g/L Mix-A, and 5 g/L glucose); Glutamate (5 g/L glutamate); Mix-A (5 g/L Mix-A); and C^−^N^−^ (lacking both carbon and nitrogen sources). Definitions: Mix-A (oligonucleotide–nucleotide mixture; oligos < 30 nt). All experiments were performed in triplicate, and results are expressed as mean ± SD. Statistical significance was determined by one-way ANOVA (** *p* < 0.01).

To investigate whether rRNA-derived oligonucleotides can function as utilizable nutrients for yeast (*K. phaffii* X-33), we used an rRNA sample extracted from *S*. *cerevisiae* and enzymatically hydrolyzed with nuclease P1 (Mix-A, provided by Nanjing Tongkai Biotechnology Co., Ltd., Nanjing, China). Mix-A contained 20–35% RNA oligonucleotides, of which > 90% were shorter than 30 nt, and 65–80% 5′-phosphate nucleotides (Figures S1 and S2, Table S1). The purity of the RNA sample exceeded 95%, with only water and salts as impurities. To minimize interference from other organic compounds (which contain both carbon and nitrogen sources), we used a basal medium containing only mineral salts (0.5 g/L K_2_HPO_4_, 0.5 g/L MgSO_4_, 0.5 g/L NaCl), with NaNO_3_ serving as the nitrogen source and control, and glucose as a carbon source when required (Figure 1).

Yeast grew well when Mix-A was used as the sole nitrogen source, although the growth rate was lower than in the YPD medium (Figure 1A). In the stationary phase, the Mix-A culture reached a maximum cell density of 3.2 × 10^9^ CFU/mL (lg ≈ 9.5), which was comparable to that achieved with glutamate (Glu). Remarkably, the growth rate with Mix-A (1.8 d^−1^) was higher than with glutamate (0.8 d^−1^) (Figure 1B). These results indicate that the mixture of RNA oligonucleotides and nucleotides can serve as an efficient nitrogen source, comparable to conventional amino acids, while an inorganic nitrogen source (NaNO_3_) supported limited growth.

Interestingly, when glucose was removed, and Mix-A provided both nitrogen and carbon sources, yeast still grew, indicating that Mix-A could partially support growth as both a nitrogen and carbon source (Figure 1C), although the carbon contribution was limited. In addition, Mix-A enhanced growth rate when added to YPD medium (from 4.1 to 5.3 d^−1^) (Figure 1D), suggesting that Mix-A can promote yeast growth in the presence of other nutrients.

Collectively, these results show that Mix-A serves as an effective nutrient source for yeast and can even enhance yeast growth in nutrient-replete media. Because Mix-A contains both RNA oligonucleotides and nucleotides, we next examined which fraction is primarily utilized by yeast and how this affects the use of other nucleic acid-derived substrates.

### 3.2. Nucleosides Can Be Efficiently Utilized by Yeast in the Presence of RNA Oligonucleotides

Nucleosides, which are widely distributed in foods and environmental matrices [3,26,27], have been regarded as the principal utilizable components of exogenous RNA for cells [6,28]. However, our results showed that the oligonucleotide–nucleotide mixture (Mix-A) also effectively supported yeast growth (Figure 1). To compare the efficiencies with which different nucleic acid substrates are utilized by yeast, and to mimic natural food matrices in which multiple RNA-derived components coexist and may influence each other’s utilization, yeast was cultured with Mix-A, nucleosides (NS), nucleotides (NT), or their combinations as the sole nitrogen source.

**Figure 2:**
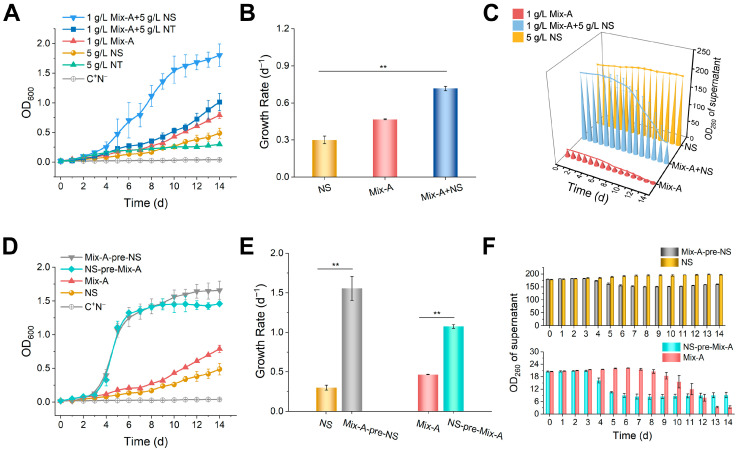
Simultaneous utilization of RNA oligonucleotides and nucleosides by yeast. (**A**–**C**) Growth in media containing Mix-A in combination with mixed nucleosides or mixed nucleotides as the sole nitrogen source. (**D**–**F**) Yeast pre-cultured in 1 g/L Mix-A or 5 g/L mixed nucleosides for 3 days (OD_600_ ≈ 0.5) was transferred into media containing 5 g/L mixed nucleosides or 1 g/L Mix-A at a 5% inoculation rate. (**A**,**D**) Growth curves. (**B**,**E**) Growth rates. (**C**,**F**) OD_260_ values of culture supernatants (after cell removal). Media compositions: 1 g/L Mix-A + 5 g/L NS: 1 g/L of Mix-A and 5 g/L of mixed nucleosides; 1 g/L Mix-A + 5 g/L NT: 1 g/L Mix-A and 5 g/L mixed nucleotides; 1 g/L Mix-A: 1 g/L Mix-A; 5 g/L NS: 5 g/L mixed nucleosides; 5 g/L NT: 5 g/L mixed nucleotides; Mix-A-pre-NS: Yeast pre-cultured in 1 g/L Mix-A transferred to 5 g/L mixed nucleosides; NS-pre-Mix-A: Yeast pre-cultured in 5 g/L mixed nucleosides transferred to 1 g/L Mix-A; C^+^N^−^: Medium lacking nitrogen source. All media contained 20 g/L glucose. Definitions: Mix-A (oligonucleotide–nucleotide mixture; oligos < 30 nt); NS (mixed nucleosides): adenosine, guanosine, cytidine, uridine, and inosine; NT (mixed nucleotides): AMP, GMP, CMP, and UMP. Data are expressed as means ± SD (n = 3). Statistical significance was determined by one-way ANOVA (** *p* < 0.01).

Yeast grew more slowly on nucleotides (NT; a mixture of AMP, GMP, CMP, and UMP) than on Mix-A. Moreover, co-supplementation with Mix-A and nucleotides (1.0 g/L Mix-A + 5.0 g/L NT) produced a growth curve only slightly higher than that of Mix-A alone (Figure 2A). These results indicate that, under Mix-A + NT conditions, nucleotides contribute minimally to overall growth, and yeast primarily utilizes the RNA oligonucleotide fraction of Mix-A.

Moreover, yeast growth on nucleosides (NS; a mixture of adenosine, guanosine, cytidine, uridine, and inosine) as the nitrogen source was also limited. Surprisingly, when a combination of 1.0 g/L Mix-A and 5.0 g/L NS was used, the culture reached a significantly higher cell density (OD_600_ = 1.8 on the 14th day), exceeding all other groups (OD_600_ < 1.0 on the 14th day) (Figure 2A). Although initial growth rates were comparable, the overall growth followed the order: Mix-A + NS > Mix-A > NS (Figure 2B). Since nucleic acid compounds exhibit maximal absorbance at 260 nm, the OD_260_ can be used as an indicator of the residual nucleic acid content in the culture medium. Although OD_260_ does not directly measure intracellular uptake, the reduction in supernatant OD_260_ serves as an indirect indicator of extracellular nucleic acid consumption. Compared with the initial (day 0) medium, a pronounced decline in the OD_260_ of more than 60% was observed in the Mix-A + NS group during prolonged cultivation (Figure 2C). This decrease suggests that both Mix-A and NS were utilized during cultivation, indicating that yeast is capable of simultaneously utilizing RNA oligonucleotides and nucleosides.

When supplied with nucleosides alone, yeast exhibited limited growth, whereas co-supplementation with Mix-A (Mix-A + NS) markedly enhanced cell proliferation (Figure 2A–C). To further explore this interaction, yeast was pre-cultured for three days (OD_600_ ≈ 0.5) in medium containing either 1.0 g/L Mix-A or 5.0 g/L NS, and then transferred (5% *v*/*v* inoculum) into fresh medium containing the alternate nitrogen source (5.0 g/L NS or 1.0 g/L Mix-A, respectively). These groups were designated as Mix-A-pre-NS and NS-pre-Mix-A, respectively. Compared with the control groups continuously cultivated in NS or Mix-A, the yeast cell densities of Mix-A-pre-NS and NS-pre-Mix-A were significantly higher (Figure 2D). On the 7th day, the yeast cell densities increased by 7–9 fold, with corresponding increases in growth rates of approximately 5-fold for Mix-A-pre-NS and 2-fold for NS-pre-Mix-A (Figure 2D,E). NS and Mix-A consumption patterns were consistent with yeast growth (Figure 2F). These results suggest that pre-culturing yeast with either RNA oligonucleotides or nucleosides enhances the utilization efficiency of both substrates, implying that they may be metabolized through shared pathways. Moreover, RNA oligonucleotides exerted a stronger promotive effect, highlighting their greater nutritional contribution.

Since Mix-A contains both RNA oligonucleotides and nucleotides, we further examined whether RNA oligonucleotides could facilitate the utilization of nucleotides by yeast. Similar experiments were conducted using nucleotides to replace nucleosides. Even after pre-culturing in Mix-A-containing medium (Mix-A-pre-NT group), no significant increase in yeast growth rate (0.3–0.4 d^−1^) was observed (Figure S3). Increasing the NT concentration to 15–25 g/L moderately improved the yeast growth rate (to 0.7 d^−1^), whereas further increases to 30–45 g/L led to a slight decline in yeast growth rate (0.6 d^−1^) (Figure S4), indicating that nucleotides are inefficient substrates for yeast.

Together, these findings indicate that RNA oligonucleotides in Mix-A are more readily utilized by yeast than nucleotides. Moreover, yeast can co-utilize RNA oligonucleotides and nucleosides, with the RNA oligonucleotides markedly enhancing nucleoside utilization. These results motivated us to further determine whether yeast can take up and utilize RNA oligonucleotides as independent substrates.

### 3.3. Yeast Preferentially Utilizes Short RNA Oligonucleotides Compared to Nucleotides

**Figure 3:**
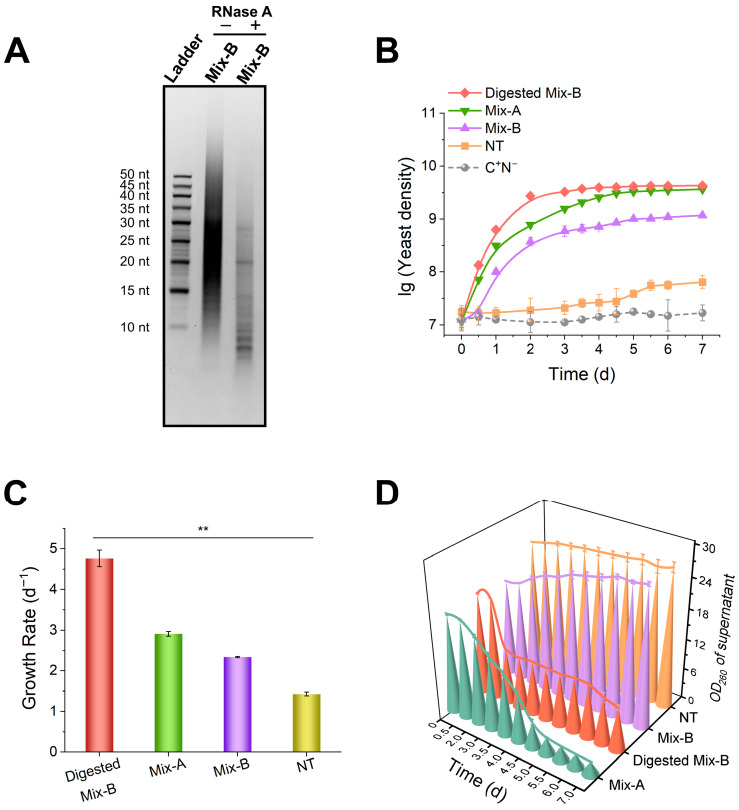
Short RNA oligonucleotides are more efficiently utilized by yeast. (**A**) 15% dPAGE analysis of RNase A digestion products. “−” indicates control group without RNase A; “+” indicates the RNase A-treated group. Digestion conditions: 1.0 g/L Mix-B, 0.01 g/L RNase A, incubated at 28 °C for 3 days. (**B**,**C**) Growth curves and growth rates under different nitrogen sources. (**D**) OD_260_ values of culture supernatants after cell removal. Media compositions: Mix-B: 1 g/L Mix-B; Digested Mix-B: 1 g/L RNase A-treated Mix-B; Mix-A: 1 g/L Mix-A; NT: 1 g/L mixed nucleotides; C^+^N^−^: medium lacking nitrogen source. All media contained 20 g/L glucose. Definitions: Mix-A (oligonucleotide–nucleotide mixture; oligos < 30 nt); Mix-B: high-purity RNA oligonucleotide preparation (10–100 nt); Digested Mix-B: RNase A-digested Mix-B (5–30 nt); NT (mixed nucleotides): AMP, GMP, CMP, and UMP. Results are expressed as means ± SD (n = 3). Statistical significance was determined by one-way ANOVA (** *p* < 0.01).

Given that yeast readily utilized the RNA oligonucleotides in Mix-A, we next examined whether yeast could also use RNA oligonucleotides supplied independently. To this end, we used Mix-B, a high-purity RNA oligonucleotide preparation (10–100 nt; > 90% oligonucleotides and <1% nucleotides) derived from *S. cerevisiae* rRNA (Figure S1). Mix-B was further digested with RNase A to generate shorter RNA oligonucleotides (5–30 nt) with 3′ phosphate groups (Figure 3A). Because RNase A does not produce nucleotides, the digestion products consisted exclusively of RNA oligonucleotides. For comparison, a mixture of nucleotides (NT; AMP, UMP, CMP, and GMP) was also used.

Yeast exhibited higher growth in all media containing RNA oligonucleotides (Mix-A, Mix-B, and Digested Mix-B) than in the nucleotide (NT) group (Figure 3B). The highest growth rate (4.8 d^−1^) was observed in the Digested Mix-B (5–30 nt) group, approximately four times that of the NT group (1.4 d^−1^). After seven days of cultivation, the maximum cell densities in the Mix-A and Digested Mix-B groups reached approximately 4.0 × 10^9^ CFU/mL (lg ≈ 9.6), nearly 100-fold higher than that of the NT group (5.0 × 10^7^ CFU/mL, lg ≈ 7.7). Among the RNA oligonucleotide groups, yeast grew fastest on Digested Mix-B (5–30 nt), followed by Mix-A (<30 nt), and slowest on Mix-B (10–100 nt) (Figure 3B,C). These results indicate that yeast utilizes RNA oligonucleotides more efficiently than nucleotides, with shorter RNA oligonucleotides being preferentially utilized.

Absorbance at 260 nm (OD_260_) was measured in the cell-free supernatant to assess RNA oligonucleotide consumption. Interestingly, OD_260_ for Mix-A and Digested Mix-B decreased by more than 80%, while little change was observed in Mix-B and NT. Notably, the OD_260_ of Mix-B even slightly increased, likely due to partial hydrolysis of longer RNA oligonucleotides (Figure 3D). These results confirm that yeast preferentially utilizes short RNA oligonucleotides (5–30 nt) over longer RNA or nucleotides. To further evaluate whether this preferential utilization is reflected at the molecular level, we examined whether short RNA oligonucleotides modulate the expression of genes involved in nucleic acid metabolism, particularly RNA-degrading nucleases.

### 3.4. Short RNA Oligonucleotides Induce Higher Expression of Yeast Nucleases

Nucleases are key enzymes in nucleic acid metabolism [29,30,31], a set of processes that are closely associated with the cellular handling and potential utilization of nucleic acid substrates. Accordingly, we examined nuclease-related gene expression (Table S2) to assess whether yeast actively utilizes RNA oligonucleotides.

**Figure 4:**
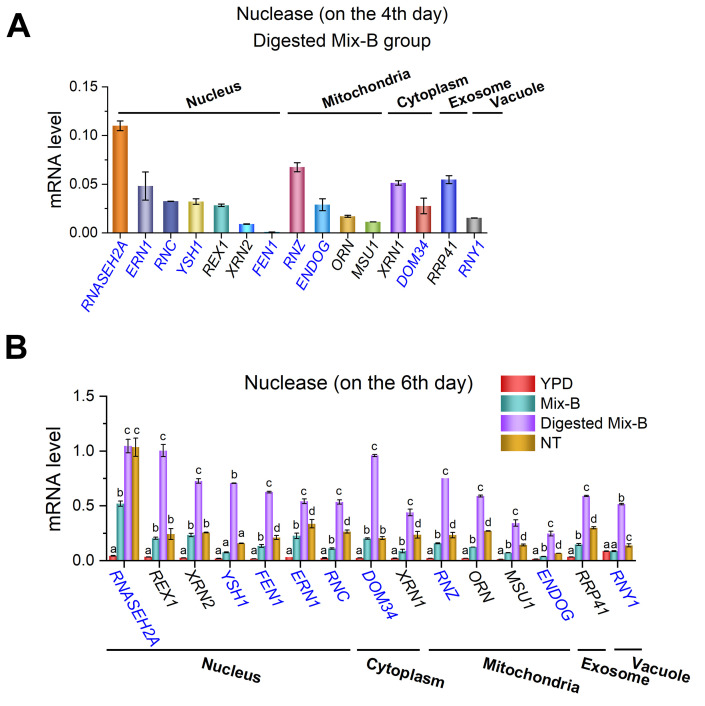
Short RNA oligonucleotides induce upregulation of the nuclease gene in yeast. (**A**) Expression levels on the 4th day in the RNase A-digested oligonucleotides (Digested Mix-B) group. (**B**) Expression levels on the 6th day in Mix-B, Digested Mix-B, NT, and YPD groups. Nucleases localized to the nucleus, cytoplasm, mitochondria, exosomes, and vacuoles were analyzed. Yeast cultured in YPD served as the control. *GAPDH* was used as the reference gene. Exonucleases are shown in black, endonucleases are shown in blue. Media compositions: YPD (5 g/L yeast extract and peptone); Mix-B (5 g/L Mix-B, 10–100 nt); Digested Mix-B (5 g/L RNase A-treated Mix-B, 5–30 nt); NT (5 g/L mixture of nucleotides (AMP, GMP, CMP, and UMP)). All media contained 20 g/L glucose. Values are expressed as mean ± SD (n = 6). Different letters (a–d) indicate significant differences (*p* < 0.05).

As shown in Figure 4A (Figure S5), mRNA levels of yeast nucleases were measured on the fourth day of cultivation, with RNase A-digested oligonucleotides (Digested Mix-B) as the sole nitrogen source. Multiple nucleases were expressed in distinct cellular compartments, reflecting the compartmentalized nature of nucleic acid metabolism in yeast. Specifically, *RNASEH2A* in the nucleus, *RNZ* in the mitochondria, *XRN1* in the cytoplasm, and *RRP41* in exosomes showed relatively high expression levels. These findings suggest that RNA oligonucleotide degradation involves coordinated actions of multiple organelles. Furthermore, short RNA oligonucleotides significantly induced nuclease gene expression, indicating that yeast engages nucleic acid degradation pathways to utilize these molecules.

Based on these results, nuclease gene expression was used as an indicator to assess the yeast’s efficiency in utilizing RNA oligonucleotides of different lengths. Three nucleic acid groups (Mix-B, Digested Mix-B, and NT) were compared with the YPD control group. Interestingly, on the sixth day of cultivation, most nucleases exhibited higher expression in all RNA-supplemented groups than in the YPD group (Figure 4B). Notably, in the RNase A-treated group (Digested Mix-B, 5–30 nt), nucleases expression (except *RNASEH2A*) was significantly higher than in both Mix-B and NT.

These findings suggest that short RNA oligonucleotides (5–30 nt) are associated with increased expression of RNA-degrading nucleases, consistent with their more efficient utilization by yeast. Although growth assays and nuclease upregulation strongly support yeast utilization of short RNA oligonucleotides, they do not provide direct visualization of cellular entry. To obtain such direct evidence, we next monitored the internalization of a fluorescently labeled RNA oligonucleotide using confocal imaging.

### 3.5. Yeast Internalizes and Utilizes Chemically Synthesized RNA Oligonucleotides

**Figure 5:**
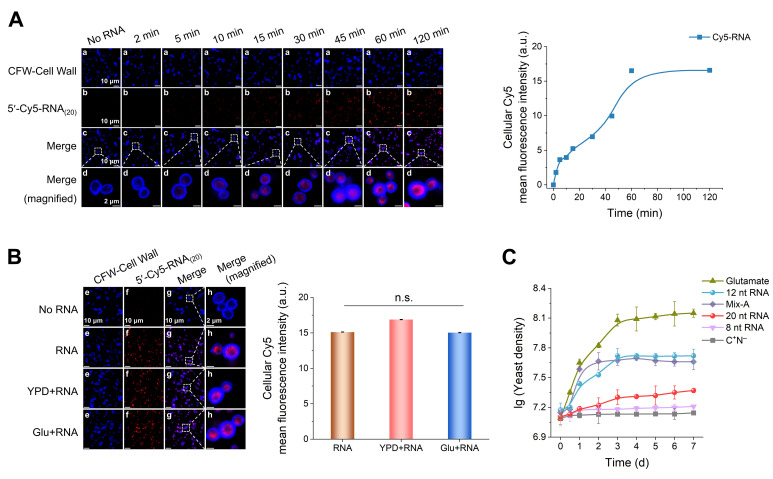
Confocal microscopy and cultivation assays showing the uptake and utilization of chemically synthesized RNA oligonucleotides by yeast. (**A**) Left panel: Time-dependent uptake of 1 μM 5′-Cy5-RNA₍_20_₎. Right panel: Quantitative analysis of intracellular Cy5 relative fluorescence intensity. (**B**) Left panel: Uptake of 5′-Cy5-RNA₍_20_₎ in the presence of additional nutrients. Yeast cells were incubated for 120 min with 1 μM 5′-Cy5-RNA₍_20_₎ alone (RNA), or together with 5 g/L yeast extract and peptone (YPD + RNA) or 5 g/L glutamate (Glu + RNA). Right panel: Quantitative analysis of intracellular Cy5 relative fluorescence intensity. (**C**) Growth curves of yeast cultured with chemically synthesized RNA oligonucleotides of different lengths (8, 12, or 20 nt; 0.8 g/L) as the sole nitrogen source. Comparison groups included 0.8 g/L Mix-A (oligonucleotide–nucleotide mixture; oligos < 30 nt), 0.8 g/L glutamate (Glu), and a nitrogen-free control (C^+^N^−^). All media contained 20 g/L glucose. Scale bars: 10 μm (panels a–c and e–g) and 2 µm (panels d and h). Fluorescence signals: CFW (cell wall, blue); 5′-Cy5–RNA_(20)_ (internalized RNA, red); Merge (overlay). Fluorescence intensity was quantified from 12 image fields across three biological replicates. Data are presented as mean ± SD (n = 3); n.s., not significant. Statistical significance was determined by one-way ANOVA.

To determine whether yeast can directly internalize RNA oligonucleotides, yeast cells were incubated with 1 μM 5′-Cy5–RNA_(20)_ (a 20 nt RNA labeled with the fluorophore Cyanine 5 (Cy5) at the 5′ terminus) for 2–120 min, with the RNA serving as the sole nitrogen source. Yeast cell walls were stained with Calcofluor White (CFW), and both CFW and Cy5 fluorescence signals were imaged using confocal laser scanning microscopy. Red Cy5 fluorescence was observed inside yeast cells as early as 5 min after incubation and progressively intensified over time (Figure 5A, left). In the enlarged merged images, Cy5 signals were clearly localized within the CFW-stained cell wall boundary (blue), confirming internalization (Figure 5A, panel d). Quantitative analysis revealed a time-dependent increase in intracellular Cy5 fluorescence intensity, reaching a plateau after approximately 60 min (Figure 5A, right), suggesting a dynamic equilibrium between RNA uptake and utilization.

To assess whether RNA uptake occurs in the presence of other nutrients, yeast was cultured for 120 min in media containing 1 μM 5′-Cy5–RNA_(20)_ supplemented with either 5 g/L yeast extract and peptone (YPD) or 5 g/L glutamate (Glu). Cy5 fluorescence was clearly detected within yeast cells under all culture conditions (Figure 5B, left). Quantitative analysis showed no significant difference in intracellular Cy5 intensity among the YPD + RNA, Glu + RNA, and RNA-only groups (*p* > 0.05; Figure 5B, right), indicating that yeast actively internalizes RNA even in the presence of other nutrients.

Next, to verify whether yeast can utilize internalized RNA oligonucleotides, we cultured yeasts in basal medium containing 0.8 g/L chemically synthesized RNAs of different lengths (8, 12, and 20 nt; sequences listed in Table S4) as the sole nitrogen source. Yeast grew in all RNA-supplemented media (Figure 5C), with the most pronounced growth observed with 12 nt RNA (lg cell density = 7.7), comparable to Mix-A (lg cell density = 7.6). These results show that yeast utilization of RNA oligonucleotides is length-dependent, with short RNA oligonucleotides (12–20 nt) more efficiently absorbed and utilized.

Together, these findings demonstrate that yeast can internalize RNA oligonucleotides and use them as nutrient sources, with short oligonucleotides being utilized more efficiently than nucleotides. To further substantiate this uptake and utilization, as well as to explore potential cellular mechanisms involved, we next performed a proteomic analysis.

### 3.6. Proteomic Analysis Reveals Key Pathways of RNA Oligonucleotide Uptake and Metabolism in Yeast

**Figure 6:**
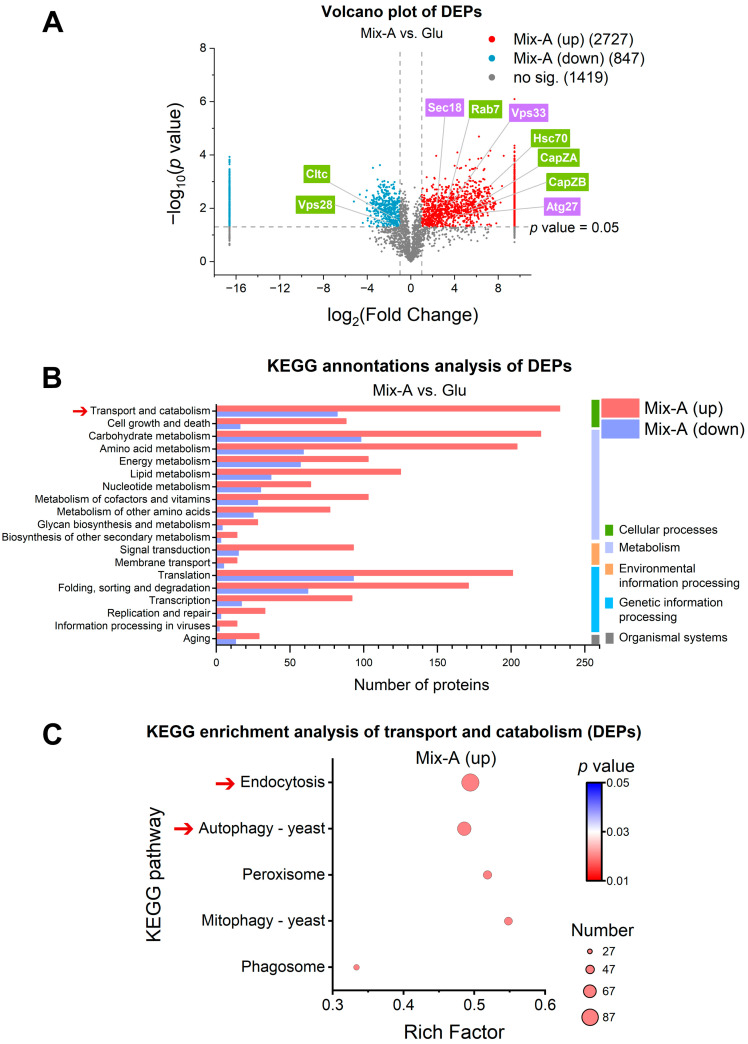
Proteomic analysis of key pathways involved in RNA oligonucleotide uptake and metabolism in yeast. (**A**) Volcano plots of differentially expressed proteins (DEPs). DEPs were selected with fold change values (FC) >2 or <0.5 and *p* < 0.05. Proteins associated with endocytosis and autophagy are highlighted in green and purple, respectively. (**B**) KEGG annotation of DEPs. (**C**) KEGG pathway enrichment analysis of DEPs involved in transport and catabolism. Red arrows indicate pathways significantly enriched among upregulated proteins in the Mix-A group, particularly those related to endocytosis and autophagy. Dot size represents the number of associated proteins. “Mix-A (up)” and “Mix-A (down)” denote proteins upregulated or downregulated in the Mix-A group, respectively. Media: Mix-A, 5 g/L Mix-A (oligonucleotide–nucleotide mixture; oligos < 30 nt) as sole nitrogen source; Glu, 5 g/L glutamate as sole nitrogen source (control group). All media contained 20 g/L glucose.

To further substantiate the utilization of RNA oligonucleotides by yeast and to explore the cellular processes associated with their uptake and metabolism, we performed a quantitative proteomic analysis comparing yeast cells grown on Mix-A with those grown on glutamate (Glu) as the control nitrogen source. A total of 2727 proteins were upregulated, and 847 were downregulated in the Mix-A group (Figure 6A), indicating that the uptake and utilization of RNA oligonucleotides activate metabolic pathways beyond those required for conventional amino acid–based nutrition. KEGG enrichment analysis further showed that 233 proteins upregulated in the Mix-A group were predominantly associated with transport and catabolic pathways (Figure 6B), with prominent enrichment in endocytosis and autophagy (Figure 6C). Because these pathways mediate the uptake and metabolism of extracellular macromolecules, and yeast preferentially utilizes the RNA oligonucleotide fraction of Mix-A (Figure 2), we hypothesized that endocytosis and autophagy may contribute to yeast utilization of RNA oligonucleotides. To explore this potential mechanism, we examined key proteins in these pathways to assess how yeast may internalize and metabolize RNA oligonucleotides. Quantitative protein abundance and statistical analyses for proteins involved in the pathways discussed below are provided in Table S5.

#### 3.6.1. Uptake via Endocytosis

In yeast, endocytosis serves as a principal pathway for the uptake of extracellular nutrients, including macromolecules such as proteins and polysaccharides [32]. In the Mix-A group, upregulated proteins were significantly enriched in endocytic pathways (Figure 6C). Notably, Hsc70 (required for clathrin uncoating) and CapZA/CapZB (involved in early endosome formation) were specifically upregulated in the Mix-A group but not detected in the Glu group (Figure S6A). Additionally, Rab7, which mediates the fusion of late endosomes with vacuoles, was expressed in both groups but at higher levels in the Mix-A group (Figure S6B).

These findings indicate that exogenous RNA oligonucleotides stimulate endocytic activity, supporting their internalization by yeast and suggesting that endocytosis may serve as a major route for their cellular uptake. As uptake represents the first step of nutrient utilization, we next examined whether internalized RNA oligonucleotides undergo intracellular catabolic processing.

#### 3.6.2. Catabolism: Autophagy, RNA Degradation, and Nucleotide Catabolism

Upregulated proteins in the Mix-A group were significantly enriched in autophagy (Figure 6C). Autophagy is a highly conserved catabolic process that enables eukaryotic cells to maintain homeostasis by degrading and recycling intracellular components [33,34]. In this study, proteins related to autophagosome formation (e.g., Atg7, Vps34, and Atg8) and the fusion of autophagosomes with vacuoles (e.g., Vti1, Vps11, and Vps18) were upregulated in the Mix-A group but were undetectable in the Glu group (Figure S7A). Additionally, several autophagy-related proteins expressed in both groups, such as Atg27 and Vps15 (autophagosome formation) and Sec18 and Vps33 (vacuole fusion), also exhibited significantly higher expression in the Mix-A group (Figure S7B). These findings suggest that RNA oligonucleotides internalized by yeast may be degraded through an autophagy-dependent pathway.

Additionally, cytoplasmic RNA-degrading enzymes, including the 5′-exonuclease Xrn1 and exosome components (e.g., Rrp46, Rrp44, and Rrp42), were significantly upregulated in the Mix-A group (Figure S7D–F), indicating enhanced RNA degradation during RNA oligonucleotide utilization. Although autophagy occurs in the vacuole and RNA-degrading enzymes are cytoplasmic, these processes likely complement each other, contributing to the efficient degradation of the internalized RNA oligonucleotides into nucleosides and nucleotides, which are subsequently routed into further catabolic pathways and diverse biosynthetic processes.

Furthermore, enzymes involved in purine and pyrimidine catabolism were significantly upregulated in the Mix-A group (*p* < 0.05; Figure S7G–J), suggesting that nucleotides and nucleosides derived from RNA oligonucleotides are further catabolized to produce *β*-alanine and ammonium (NH_4_^+^) through nucleotide catabolic pathways. These metabolites serve as versatile precursors that support the synthesis of amino acids and a broad range of other biomolecules.

Taken together, these results demonstrate that internalized RNA oligonucleotides are actively catabolized through coordinated autophagy, RNA degradation, and nucleotide catabolism pathways, generating nucleotides, nucleosides, *β*-alanine, and ammonium (NH_4_^+^) that are subsequently funneled into downstream anabolic pathways.

#### 3.6.3. Anabolism: Nucleotides, Glycerophospholipids, and Amino Acids Biosynthesis

To determine whether the catabolic products of RNA oligonucleotides are reintegrated into anabolic pathways, we examined key enzymes involved in nucleotide, amino acid, and glycerophospholipid biosynthesis, which collectively represent central routes of nitrogen metabolism—a core foundation of anabolic processes.

Salvage-pathway enzymes for nucleotide synthesis were highly expressed in the Mix-A group (Figure S8), indicating efficient reuse of RNA oligonucleotide-derived products for nucleotide synthesis. Interestingly, enzymes related to de novo purine synthesis were downregulated in the Mix-A group (Figure S8A,B), likely due to tight regulation of the purine nucleotide pool, which can be maintained through the salvage pathway. However, key enzymes involved in both de novo and salvage pyrimidine synthesis were upregulated in the Mix-A group (Figure S8C,D), suggesting a higher metabolic demand for pyrimidine nucleotides, which are critical for nucleic acid synthesis and serve as precursors for other metabolites. Notably, CTP, a pyrimidine nucleotide, serves as a crucial substrate for glycerophospholipid biosynthesis, a pathway essential for membrane formation [35,36]. Consistently, enzymes involved in glycerophospholipid synthesis, such as Cdipt, Ept1, and Cpt1, were significantly upregulated in the Mix-A group (Figure S9A–C), indicating that RNA oligonucleotide-derived metabolites also contribute to membrane lipid biosynthesis.

Because amino acids are fundamental biomolecules required for cellular growth and metabolism, we also examined the key biosynthetic enzymes involved in their synthesis. Glutamine synthetase (GlnA) and glutamate synthase (Glt1) were significantly upregulated in the Mix-A group. In addition, 4-aminobutyrate aminotransferase (Puue) was also markedly elevated, catalyzing the conversion of *β*-alanine (from pyrimidine degradation) to glutamate, thus linking RNA catabolism to amino acid biosynthesis—a previously underappreciated metabolic pathway (Figure S9D,E). These findings suggest that NH_4_^+^ and *β*-alanine generated from RNA oligonucleotides are assimilated into glutamate and glutamine, which act as central nitrogen donors for other amino acids.

Collectively, these results demonstrate that catabolic products of RNA oligonucleotides are reintegrated into anabolic pathways, supplying precursors for nucleotide, membrane lipid, and amino acid biosynthesis. These findings reveal that RNA oligonucleotides act as bioavailable nutrients that sustain biosynthetic processes essential for cellular growth.

**Figure 7:**
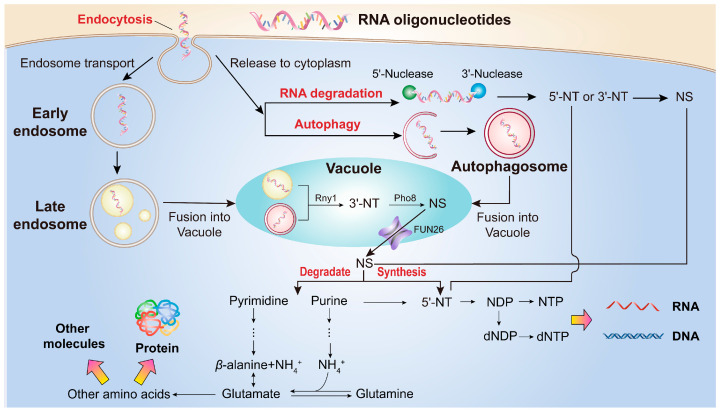
Proposed model of RNA oligonucleotide uptake and metabolism in yeast. Yeast cells internalize RNA oligonucleotides (approximately 5–30 nt) via endocytosis. Some oligonucleotides are transported through endosomes to vacuoles for degradation into nucleosides (NS), while others are released into the cytoplasm. In the cytoplasm, RNA oligonucleotides are degraded by autophagy and cytoplasmic nucleases into nucleosides and nucleotides (NT). These degradation products are subsequently utilized for RNA/DNA synthesis or further catabolized into NH_4_^+^ and *β*-alanine for the biosynthesis of amino acids and other biomolecules. Processes highlighted in red represent pathways for which associated proteins were significantly upregulated in the Mix-A group, as revealed by proteomic analysis. These pathways include endocytosis (KEGG pathway ID: map04144), autophagy (KEGG pathway ID: map04138), RNA degradation (KEGG pathway ID: map03018), purine metabolism (KEGG pathway ID: map00230), and pyrimidine metabolism (KEGG pathway ID: map00240). Quantitative proteomic data supporting the highlighted pathways are provided in Supplementary Figures S6–S9 and Table S5.

Overall, the proteomic data support the view that yeast internalizes and metabolizes RNA oligonucleotides as nutrient sources at the cellular level. These findings also inform a potential model for how RNA oligonucleotides may be taken up and metabolized in yeast, which is outlined in Figure 7.

## 4. Discussion

rRNA constitutes nearly 80% of total cellular RNA [19] and is readily fragmented by endogenous and environmental nucleases [20,21]. Consequently, rRNA-derived oligonucleotides likely represent the most prevalent nucleic acid fragments in natural ecosystems and food environments. However, most existing studies have focused on the physiological functions of nucleosides or nucleotides, whereas the nutritional fate of the predominantly present rRNA-derived oligonucleotides remains largely unexplored. In particular, it is unclear whether eukaryotic cells can directly utilize RNA oligonucleotides as nutrient sources.

In this study, we demonstrate that short rRNA-derived oligonucleotides serve as effective nutrient sources that support robust yeast growth. Proteomic analysis further showed that yeast internalizes RNA oligonucleotides and metabolizes them through a coordinated pathway, involving endocytosis for uptake, autophagy and RNA degradation for catabolism, and subsequent channeling of the resulting metabolites into anabolic processes. Collectively, these findings highlight the nutritional value of rRNA-derived oligonucleotides and advance current understanding of non-traditional nutrient sources in eukaryotic systems, particularly those relevant to food and fermentation processes.

### 4.1. rRNA-Derived Oligonucleotides as a Potential Nitrogen-Rich Nutritional Component

Nucleic acids contain a high proportion of nitrogen (15–17%), comparable to that of proteins (14–18%), suggesting their nutritional potential. Consistent with this, rRNA-derived oligonucleotides supported robust yeast growth when provided as the sole nitrogen source, reaching levels comparable to those supported by glutamic acid (Figure 1A). Because rRNA constitutes the majority of cellular RNA and undergoes substantial fragmentation during food processing and storage, rRNA-derived oligonucleotides are likely abundant in food matrices and natural environments. Given their widespread occurrence and demonstrated usability by yeast, RNA oligonucleotides may represent readily bioavailable nitrogen-rich nutrients within food systems and microbial ecosystems.

### 4.2. Preferential Utilization of Short RNA Oligonucleotides by Yeast

Interestingly, yeasts cultured on short RNA oligonucleotides (approximately 5–30 nt) exhibited higher growth efficiency than those grown on nucleotides (Figure 3), indicating that yeast preferentially utilizes short RNA fragments. Consistent with this, culturing yeast on short RNA oligonucleotides induced markedly higher expression of multiple nuclease genes than nucleotides (Figure 4), providing molecular evidence that intracellular RNA-degrading pathways are more strongly activated when short RNA fragments are available. This differential induction supports the superior bioavailability of RNA oligonucleotides compared with nucleotides. Notably, nucleases are known to serve multiple biological functions beyond nutrient utilization. In addition to processing and potential utilization of nucleic acid substrates, nucleases also play important protective roles by degrading potentially harmful exogenous RNAs, such as viral RNAs, thereby limiting their biological activity within the cell. These dual functions may jointly contribute to the elevated nuclease expression observed under RNA oligonucleotide–rich conditions. Therefore, the upregulation of nucleases in response to short RNA oligonucleotides likely reflects both enhanced nutrient utilization and a broader cellular defense strategy.

Short RNA oligonucleotides are likely to be generated widely during food processing and storage [20,21,22]. Their frequent occurrence in food systems and natural environments raises an important implication: short RNA oligonucleotides may function not only as nutrient sources but also as nutritional signals. When short RNA oligonucleotides are present at sufficient levels, likely reflecting a nutrient-rich food environment, they may trigger the expression of genes involved in nutrient metabolism, such as nucleases, thereby enabling yeast to utilize available resources more efficiently. Such regulatory responsiveness gives short RNA oligonucleotides a nutritional signaling role beyond their chemical composition.

The observation that short RNA oligonucleotides are more readily utilized than nucleotides can also be interpreted in light of the intrinsic metabolic regulation of nucleotide homeostasis. Because 5′-nucleotides are essential for genetic processes and energy metabolism, their intracellular levels remain tightly controlled [37]. Consistently, excessive nucleotide supplementation (>25 g/L) led to yeast growth inhibition (Figure S4B). Alternatively, RNA oligonucleotides cannot directly participate in nucleotide-dependent pathways but can be gradually degraded into nucleosides or 3′-nucleotides, which are subsequently converted into 5′-nucleotides when required, thereby avoiding metabolic imbalance and providing a more flexible and metabolically safer nutrient source. From an ecological perspective, directly utilizing short RNA oligonucleotides may be more energy-efficient than secreting nucleases to degrade long RNA into nucleotides.

Analogous phenomena have been observed in mammals, where dietary microRNAs (~21 nt) are absorbed by gastric epithelial cells via the SIDT1-mediated uptake and subsequently distributed via exosomes [14]. Moreover, microRNAs have been detected in human milk [38] and may be transmitted to infants via exosomes, influencing infant metabolism and gut development [39,40]. Together, these studies reinforce our finding that short RNA oligonucleotides can be internalized and utilized by yeast and further suggest that they may also represent a metabolically usable nutrient source across diverse eukaryotic systems.

### 4.3. RNA Oligonucleotides Facilitate Efficient Utilization of Nucleosides in Yeast

Nucleosides have traditionally been regarded as the primary utilizable components of extracellular RNA [2]. Unexpectedly, yeast utilized nucleosides only poorly when supplied alone, whereas the presence of RNA oligonucleotides markedly enhanced nucleoside utilization (Figure 2A–C). Furthermore, pre-cultivation with either RNA oligonucleotides or nucleosides increased the subsequent utilization of both substrates (Figure 2D–F). These observations can be explained by the fact that both RNA oligonucleotides and nucleosides are metabolized through shared pathways. Once internalized, RNA oligonucleotides are degraded into nucleosides, which become indistinguishable from exogenous nucleosides. Both substrates subsequently enter the common downstream catabolic and anabolic routes. Thus, pre-exposure to either substrate likely primes these pathways, enhancing the utilization of both.

Additionally, RNA oligonucleotides enhanced nucleoside utilization more effectively than nucleosides themselves (Figure 2D–F). This effect may be partly attributed to the ability of RNA oligonucleotides to more effectively activate cellular pathways involved in the uptake and metabolism of nucleic acid-derived substrates, including nucleosides. This interpretation is supported by our proteomic analysis, which revealed substantial upregulation of key enzymes associated with nucleic acid metabolic processes in response to RNA oligonucleotides (Figures S7–S9).

Although RNA oligonucleotides markedly enhanced nucleoside utilization, their effect on promoting nucleotide utilization was relatively limited (Figure S3). This difference may be partly attributed to the presence of negatively charged phosphate groups in nucleotides, which reduce membrane permeability and thereby restrict cellular uptake [41], whereas nucleosides can be efficiently transported via specific nucleoside transporters in yeast cells [42]. In addition, yeast growth was relatively slow during the initial cultivation period when nucleosides or RNA oligonucleotides were supplied as the sole nitrogen source (Figure 2D). This phenomenon may reflect a metabolic adaptation phase during early cultivation, in which yeast cells prioritize the induction of pathways required for the utilization of nucleic acid-derived substrates rather than rapid growth, thereby giving rise to an initial lag phase.

Together, these findings extend the prevailing view that exogenous RNA is primarily utilized only after complete degradation into nucleosides [2,43,44]. Our results demonstrate that short RNA oligonucleotides can be directly internalized and utilized by yeast and further facilitate the utilization of nucleosides.

### 4.4. Mechanistic Insights from Proteomics: Uptake, Catabolism, and Anabolism of RNA Oligonucleotides in Yeast

Proteomic analysis further substantiated that yeast utilizes RNA oligonucleotides as nutrient sources and provided mechanistic insight into pathways mediating their uptake and metabolism. When Mix-A served as the sole nitrogen source, multiple endocytosis-related proteins were markedly upregulated (Figure S6), indicating activation of endocytic pathways by the RNA oligonucleotide fraction of Mix-A. This is consistent with reports that diverse extracellular RNAs, including microRNAs, siRNAs, and mRNAs, enter eukaryotic cells primarily through endocytosis [45]. Taken together with previous reports, our results suggest that yeast may recognize extracellular RNA oligonucleotides as utilizable macromolecules and internalize them through clathrin-mediated endocytosis.

Furthermore, key proteins associated with autophagy, RNA degradation, and nucleotide catabolic pathways were also highly expressed when yeast was cultured on Mix-A (Figure S7). These results demonstrate that the internalized RNA oligonucleotide fraction of Mix-A is actively catabolized through coordinated pathways, generating nucleotides, nucleosides, *β*-alanine, and ammonium (NH_4_^+^). It is plausible that rRNA-derived oligonucleotides behave similarly to conventional nutrients, being progressively processed into bioavailable substrates rather than triggering stress or immune-like responses. Thus, RNA oligonucleotides hold functional value as readily metabolizable nutrient sources.

Additionally, the elevated expression of enzymes associated with amino acid and phospholipid biosynthesis in the Mix-A group (Figure S9) suggests that the RNA oligonucleotide fraction contributes to broader anabolic metabolism beyond nucleotide regeneration. These results indicate that RNA oligonucleotides taken up by yeast are not treated as metabolic waste but are actively incorporated into essential biosynthetic pathways. This metabolic flexibility likely results from the nitrogen-rich nature of RNA, which provides a substantial nitrogen supply compared with conventional nutrients [2,46]. Moreover, nitrogen is an essential but often limiting element for cellular growth, and nitrogen deprivation can induce autophagy to recycle endogenous RNA [31,47,48]. Our findings extend this concept by demonstrating that eukaryotic cells not only recycle endogenous RNA but also actively internalize and utilize exogenous RNA oligonucleotides to alleviate nitrogen limitation, thereby broadening the nutritional significance of RNA oligonucleotides in biological and food systems.

Based on the observation that yeast efficiently utilizes short RNA oligonucleotides as nutrient sources and the proteomic analysis showing induction of key pathways involved in nutrient uptake and metabolism, we propose a mechanistic model describing how yeast takes up and metabolizes RNA oligonucleotides (Figure 7). In this model, extracellular rRNA-derived oligonucleotides are first internalized through clathrin-mediated endocytosis. Once internalized, these RNA oligonucleotides undergo stepwise degradation via autophagy, RNA decay pathways, and nucleotide catabolism, generating metabolites such as nucleotides, nucleosides, *β*-alanine, and ammonium (NH_4_^+^). These metabolites subsequently enter anabolic pathways, supporting the synthesis of nucleic acids, proteins, phospholipids, and other essential biomolecules.

This study demonstrates that rRNA-derived oligonucleotides (approximately 5–30 nt) can be efficiently utilized as a nutrient source by the eukaryotic model organism *K. phaffii* X-33 (yeast). Our findings indicate that their uptake and metabolic processing involve coordinated activation of endocytic, autophagic, and RNA degradation pathways. These results suggest that rRNA-derived oligonucleotides represent previously underappreciated, bioavailable nutrient sources.

Previous studies have primarily focused on the utilization of short DNA and RNA oligonucleotides in prokaryotic systems, including *Escherichia coli* [49] and *Bacillus subtilis* [50]. Notably, this study provides systematic evidence for RNA oligonucleotide utilization in a eukaryotic microorganism, thereby extending previous observations that were limited to prokaryotic systems. Whether similar mechanisms operate in other eukaryotic systems, particularly in higher eukaryotic cells, remains to be explored in future studies, including the identification of uptake mechanisms, metabolic pathways, and associated physiological consequences.

From an applied perspective, the observation that short RNA oligonucleotides are more efficiently utilized than nucleotides suggests potential implications for the design of nucleotide-fortified functional foods (e.g., infant formula and FSMPs) by incorporating RNA oligonucleotides. These findings also provide a conceptual basis for the development of RNA oligonucleotide-based fermentation processes, animal feed formulations, and functional foods, highlighting the potential application of rRNA-derived oligonucleotides as novel nutrient sources or bioactive components in food science and bioprocessing.

## 5. Conclusions

This study demonstrates that short rRNA-derived oligonucleotides (approximately 5–30 nt) can serve as efficient and bioavailable nutrient sources for the eukaryotic model organism *K. phaffii X-33* (yeast). Proteomic analysis indicates that the uptake and subsequent metabolism of RNA oligonucleotides involve coordinated endocytic, autophagic, and RNA-catabolic pathways, which collectively channel their breakdown products into essential biosynthetic processes. These findings provide evidence that rRNA-derived oligonucleotides, which are widely present in food and natural environments, represent bioavailable nutrient sources for yeast cells and may have potential nutritional relevance for other eukaryotic systems. Overall, this work provides a conceptual basis for exploring RNA oligonucleotide-based functional foods and suggests potential applications in microbial fermentation, single-cell protein production, and synthetic biology.

## Data Availability

The original contributions presented in this study are included in the article/Supplementary Materials. Further inquiries can be directed to the corresponding authors.

## Supplementary Materials

The following supporting information can be downloaded at: https://www.mdpi.com/article/10.3390/foods15020318/s1, Figure S1: Composition analysis of Mix-A and Mix-B; Figure S2: Electrophoretic analysis of Mix-A by 15% denaturing PAGE (dPAGE); Figure S3: Yeast growth using RNA oligonucleotides and nucleotides as nitrogen sources; Figure S4: Effects of nucleoside and nucleotide concentrations on yeast growth; Figure S5: Nuclease gene expression in yeast cultured with RNA oligonucleotides of different lengths on day 4; Figure S6: Expression profiles of proteins associated with endocytosis; Figure S7: Expression profiles of proteins involved in autophagy, RNA degradation, and nucleotide catabolism; Figure S8: Expression profiles of proteins involved in nucleotide synthesis pathways; Figure S9: Expression profiles of proteins involved in glycerophospholipid and amino acid biosynthesis; Table S1: Purity analysis of Mix-A and Mix-B; Table S2: Information on intracellular nuclease genes in *K*. *phaffii* X-33; Table S3: Primer sequences for RT-qPCR analysis of intracellular nuclease genes in *K*. *phaffii* X-33; Table S4: Chemically synthesized RNA sequences used for the cultivation of *K. phaffii* X-33. Table S5: Quantitative proteomic analysis of proteins involved in RNA oligonucleotide uptake and metabolism in yeast.

## Abbreviations

rRNA	Ribosomal RNA
NT	Nucleotide
NS	Nucleoside
FSMPs	Foods for Special Medical Purposes
