# Supplementary material for: Yeast Efficiently Utilizes Ribosomal RNA-Derived Oligonucleotides as Bioavailable Nutrient Sources
Source: Foods. 2026 Jan 15;15(2):318. doi: 10.3390/foods15020318 (PMC12841064; doi:10.3390/foods15020318)
Supplement: Supplementary file 1 [file foods-15-00318-s001.zip › Supplementary Information.pdf]

foods

Supplementary Information

Yeast Efficiently Utilizes Ribosomal RNA-Derived Oligonucleotides as Bioavailable Nutrient Sources

Xinmei Du [1], Qitao Chen [1], Jingyun Zhuang [1], Mingqi Zhao [1], Yixin Duan [1], Shuang Wang [1] and Ran An [1,2,*], Xingguo Liang [1,2,*]

[1] College of food science and engineering, Ocean University of China, Qingdao, 266404, China
[2] Laboratory for Marine Drugs and Bioproducts, Qingdao Marine Science and Technology Center, Qingdao, 266237, China

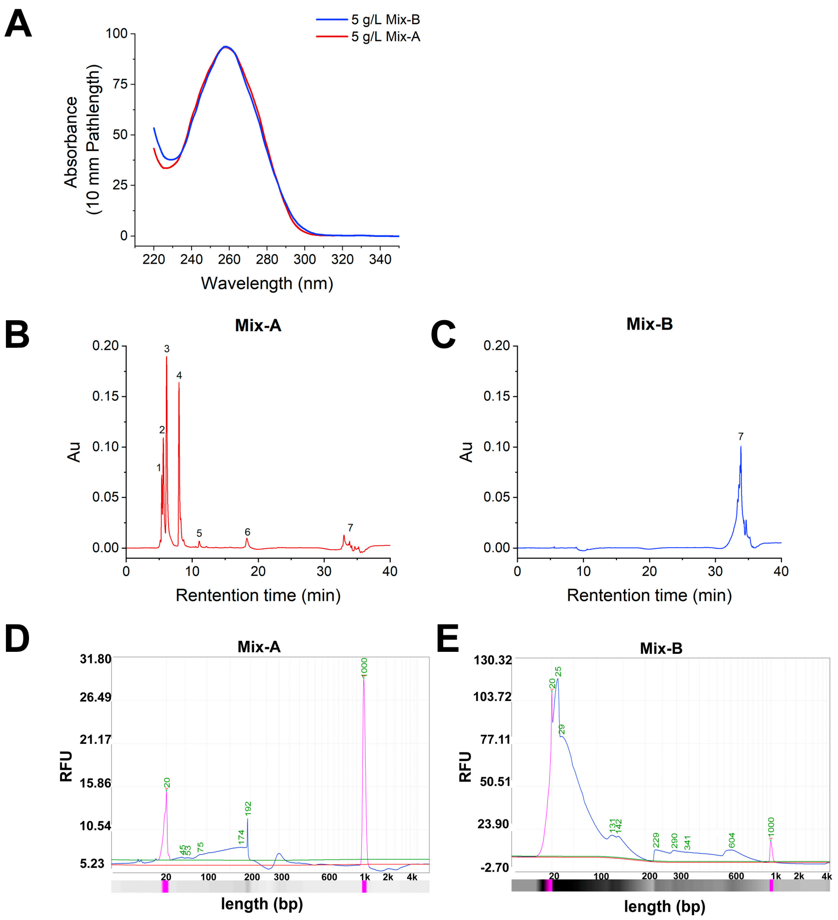

Figure S1. Composition analysis of Mix-A and Mix-B. (**A**) UV absorption spectra of Mix-A and Mix-B. (**B**, **C**) HPLC chromatograms. Peaks 1–4 correspond to CMP, UMP, GMP, and AMP, respectively, while peaks 5–7 represent RNA oligonucleotides of varying lengths. Mobile phase A: 50 mM ammonium formate aqueous solution (pH 6.45). Mobile phase B: 50% acetonitrile aqueous solution containing 50 mM ammonium formate (pH 6.45). The gradient elution was programmed as follows: 0–8 min, 10% B; 8–9 min, 10–12% B; 9–19 min, 12% B; 19–24 min, 12–20% B; 24–29 min, 20–100% B; 29–39 min, 100% B; 39–40 min, 100–10% B; 40–70 min, 10% B. The flow rate was 0.5 mL/min at 25°C, and detection was performed at 260 nm. (**D**, **E**) Capillary electrophoresis profiles (20 nt and 1000 nt markers).

https://doi.org/10.3390/foods15020318

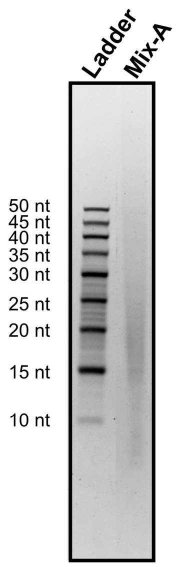

Figure S2. Electrophoretic analysis of Mix-A by 15% denaturing PAGE (dPAGE).

https://doi.org/10.3390/foods15020318

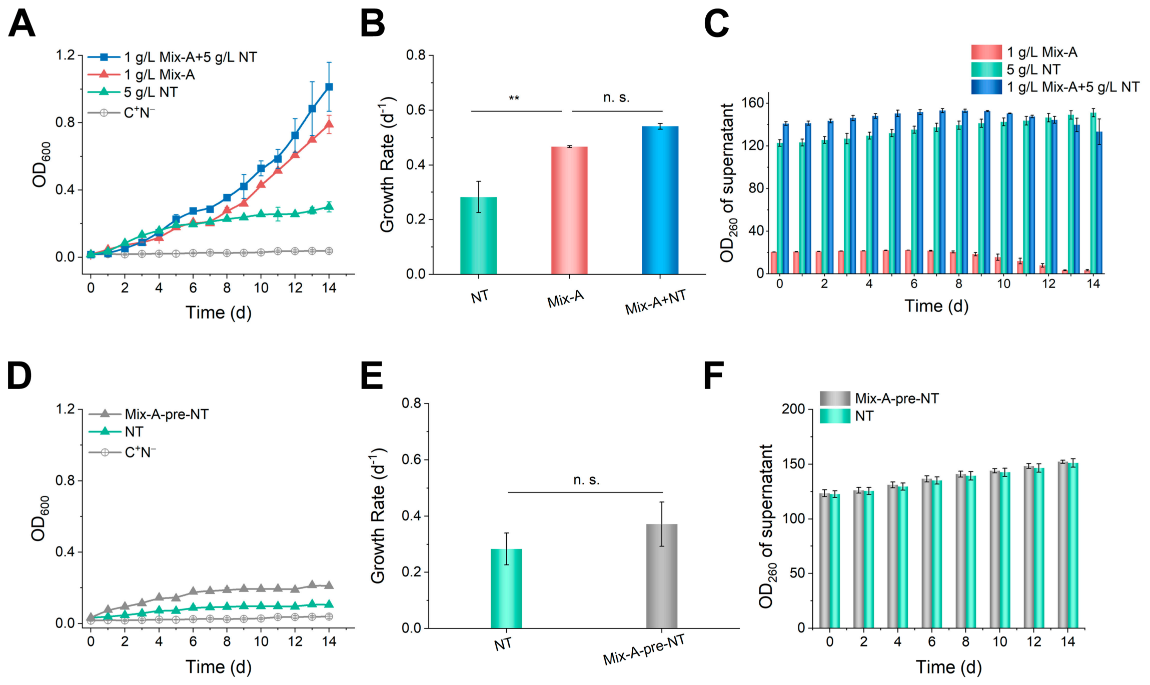

Figure S3. Yeast growth using RNA oligonucleotides and nucleotides as nitrogen sources. (**A**–**C**) Growth in media containing Mix-A and mixed nucleotides (NT) as the sole nitrogen source. (**D**–**F**) Yeast cells pre-cultured in 1 g/L Mix-A medium for 3 days (OD_{600} ≈ 0.5) were transferred into media containing 5 g/L NT at a 5% inoculation rate (Mix-A-pre-NT). (**A**, **D**) Growth curves. (**B**, **E**) Growth rates. (**C**, **F**) OD_{260} values of culture supernatants (after cell removal). Media compositions: 1 g/L Mix-A + 5 g/L NT: 1.0 g/L Mix-A and 5.0 g/L mixed nucleotides; 1 g/L Mix-A: 1.0 g/L Mix-A; 5 g/L NT: 5.0 g/L mixed nucleotides; Mix-A-pre-NT: Yeast cells transferred from 1.0 g/L Mix-A medium to 5.0 g/L mixed nucleotide medium; C^+N^-: Medium lacking any nitrogen source. All media contained 20 g/L glucose. Definitions: Mix-A (oligonucleotide–nucleotide mixture; oligos < 30 nt); NT (mixed nucleotides): AMP, GMP, CMP, and UMP. Data are expressed as means ± SD (n = 3). Statistical significance was determined by one-way ANOVA ($^{**}p < 0.01$; n.s., not significant).

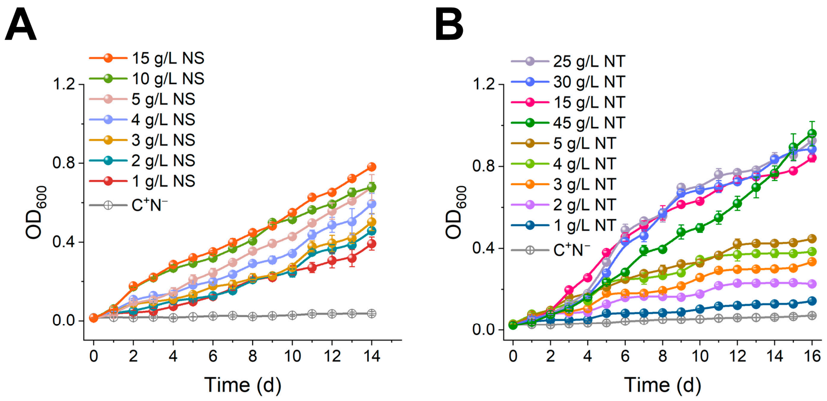

Figure S4. Effects of nucleoside and nucleotide concentrations on yeast growth. (**A**) Growth curves of yeast cultured with different concentrations of nucleosides (NS; containing adenosine, guanosine, cytidine, uridine, and inosine; 1–15 g/L) as the sole nitrogen source. (**B**) Growth curves of yeast cultured with different concentrations of nucleotides (NT; containing AMP, GMP, CMP, and UMP; 1–45 g/L) as the sole nitrogen source. All media contained 20 g/L glucose.

https://doi.org/10.3390/foods15020318

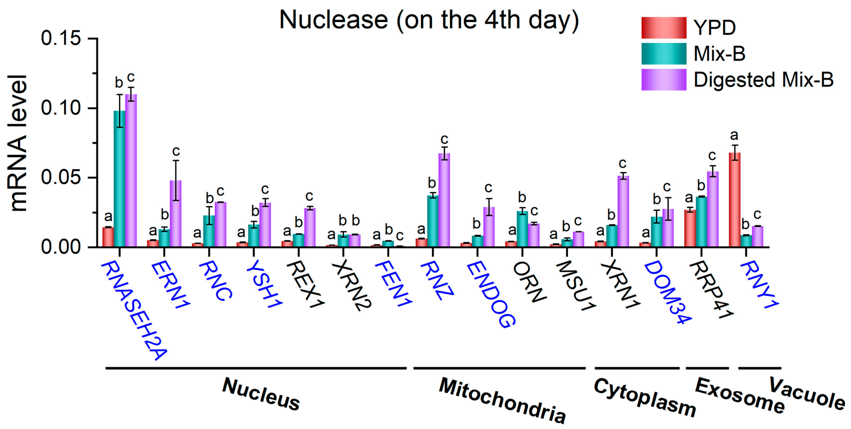

Figure S5. Nuclease gene expression in yeast cultured with RNA oligonucleotides of different lengths on day 4. Nucleases located in the nucleus, cytoplasm, mitochondria, exosomes, and vacuoles were analyzed. Yeast grown in YPD medium served as the control. Glyceraldehyde-3-phosphate dehydrogenase (*GAPDH*) was used as the reference gene for normalization. Nuclease names in black indicate exonucleases, while those in blue indicate endonucleases. YPD: 5 g/L yeast extract and peptone; Mix-B: 5 g/L oligonucleotides (10−100 nt); Digested Mix-B: 5 g/L RNase A-treated Mix-B (5−30 nt). All media contained 20 g/L glucose. Values are expressed as mean ± SD (n=6). Different letters (a–d) denote significant differences in nuclease gene expression levels ($p < 0.05$).

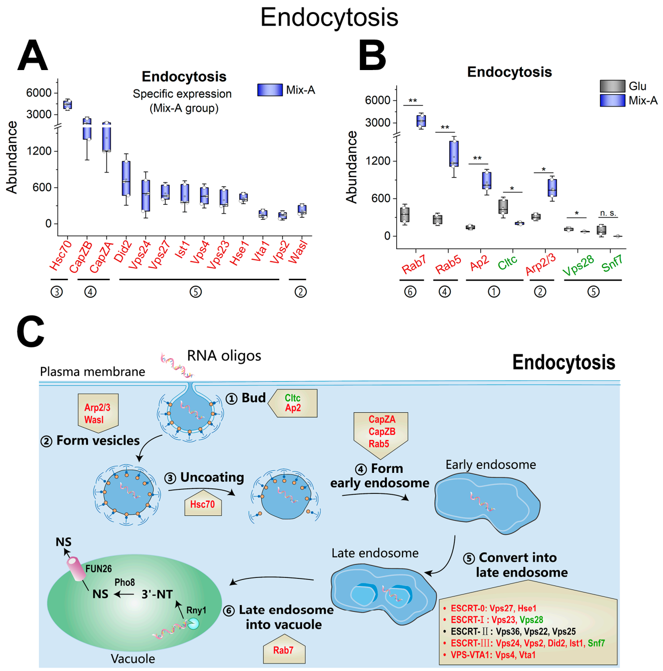

Figure S6. Expression profiles of proteins associated with endocytosis. (**A**) Proteins specifically expressed in the Mix-A group. (**B**) Proteins co-expressed in the Mix-A and glutamate (Glu) groups. (**C**) Schematic illustration of key proteins involved in the endocytic process and their relative expression levels. Proteins with higher expression in Mix-A group are indicated in red font, those with lower expression in green, and undetected proteins in black. Mix-A: 5 g/L Mix-A (oligonucleotide–nucleotide mixture; oligos < 30 nt) as the sole nitrogen source; Glu: 5 g/L glutamate as the sole nitrogen source (control group). All media contained 20 g/L glucose. Statistical significance was determined by one-way ANOVA ($^{*}p < 0.05$; $^{**}p < 0.01$; n.s., not significant). Quantitative protein abundance information (Mix-A vs. Glu) for the proteins highlighted in this figure is provided in Table S5.

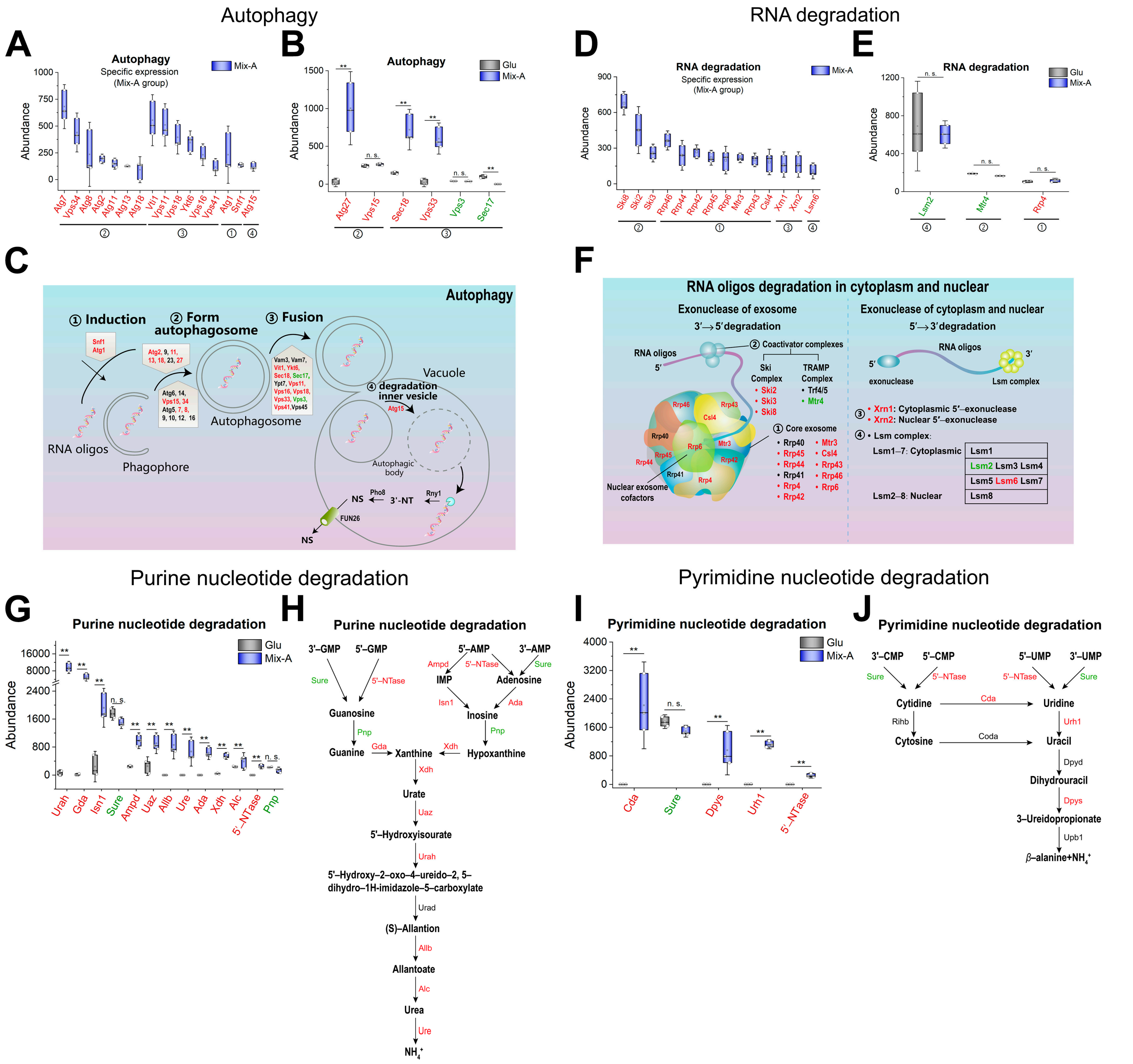

Figure S7. Expression profiles of proteins involved in autophagy, RNA degradation, and nucleotide catabolism. (**A**, **B**) Protein levels associated with autophagy. (**D**, **E**) Protein levels associated with RNA degradation. (**G**, **I**) Protein levels associated with purine and pyrimidine nucleotide catabolism, respectively. (**A**, **D**) Proteins specifically expressed in the Mix-A group. (**B**, **E**) Proteins co-expressed in the Mix-A and glutamate (Glu) groups. (**C**, **F**, **H**, **J**) Schematic illustrations of key proteins involved in autophagy, RNA degradation, and purine and pyrimidine nucleotide degradation, respectively. Proteins with higher expression in the Mix-A group are shown in red, those with lower expression in green, and undetected proteins in black. Mix-A: 5 g/L Mix-A (oligonucleotide–nucleotide mixture; oligos < 30 nt) as the sole nitrogen source; Glu: 5 g/L glutamate as the sole nitrogen source. All media contained 20 g/L glucose. Statistical significance was determined by one-way ANOVA ($^{*}p < 0.05$; $^{**}p < 0.01$; n.s., not significant). Quantitative protein abundance information (Mix-A vs. Glu) for the proteins highlighted in this figure is provided in Table S5.

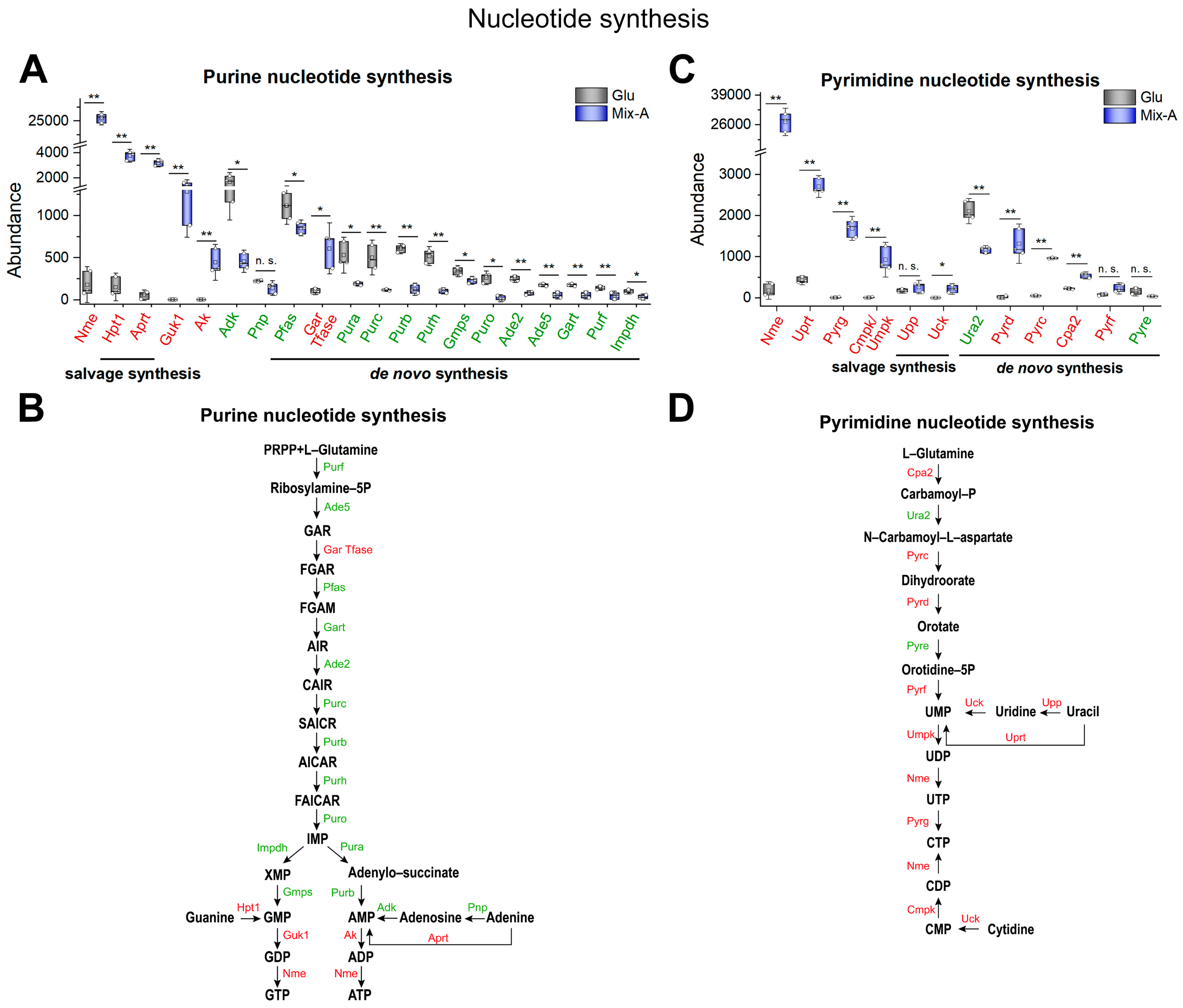

Figure S8. Expression profiles of proteins involved in nucleotide synthesis pathways. (**A**, **C**) Protein levels associated with purine and pyrimidine nucleotide synthesis. (**B**, **D**) Schematic illustrations of key proteins involved in these pathways. Proteins with higher expression in the Mix-A group are shown in red, those with lower expression in green, and undetected proteins in black. Mix-A: 5 g/L Mix-A (oligonucleotide–nucleotide mixture; oligos < 30 nt) as the sole nitrogen source; Glu: 5 g/L glutamate as the sole nitrogen source. All media contained 20 g/L glucose. Statistical significance was determined by one-way ANOVA ($^{*}p < 0.05$; $^{**}p < 0.01$; n.s., not significant). Quantitative protein abundance information (Mix-A vs. Glu) for the proteins highlighted in this figure is provided in Table S5.

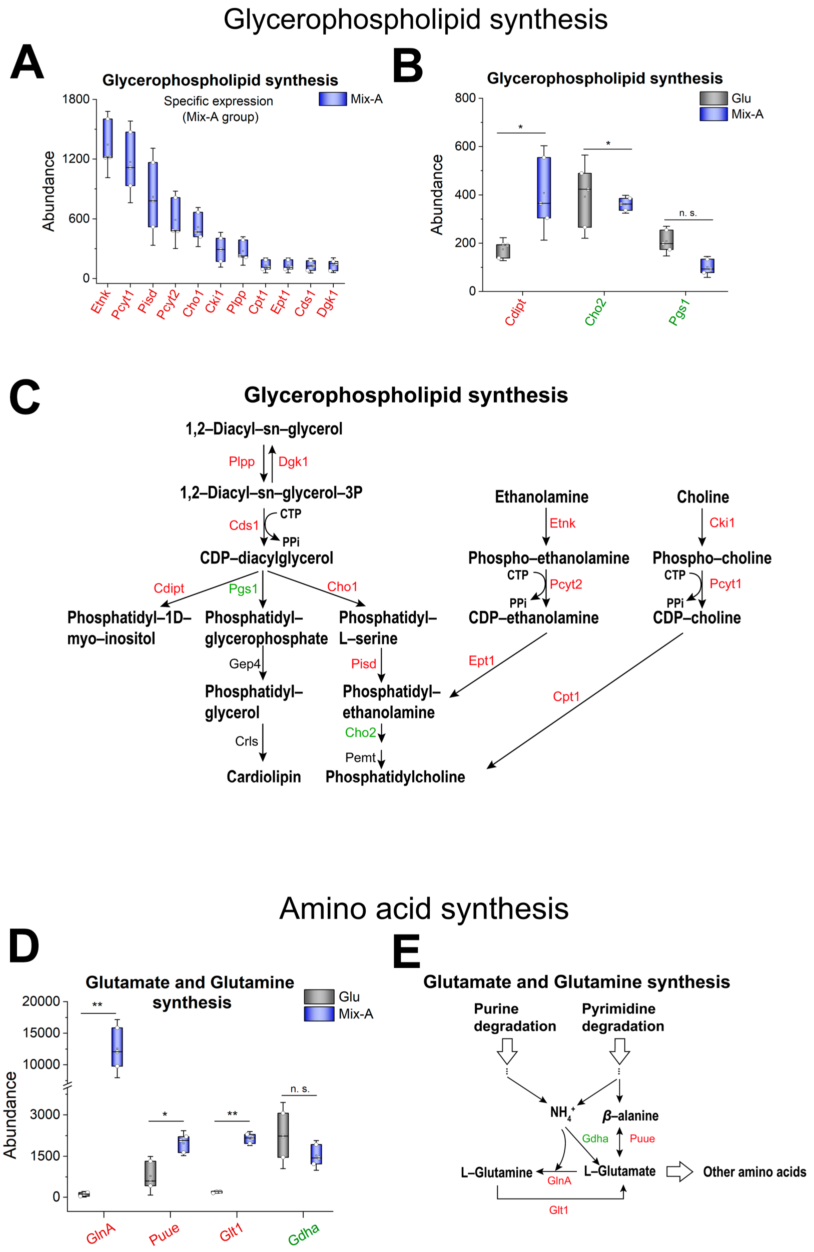

Figure S9. Expression profiles of proteins involved in glycerophospholipid and amino acid biosynthesis. (**A**, **B**) Protein levels associated with glycerophospholipid biosynthesis. (**D**) Protein levels associated with amino acid biosynthesis. (**A**) Proteins specifically expressed in the Mix-A group. (**B**) Proteins co-expressed in the Mix-A and glutamate (Glu) groups. (**C**, **E**) Schematic illustrations of key proteins involved in glycerophospholipid and amino acid biosynthetic pathways. Proteins with higher expression in the Mix-A group are shown in red, those with lower expression in green, and undetected proteins in black. Mix-A: 5 g/L Mix-A (oligonucleotide–nucleotide mixture; oligos < 30 nt) as the sole nitrogen source; Glu: 5 g/L glutamate as the sole nitrogen source. All media contained 20 g/L glucose. Statistical significance was determined by one-way ANOVA ($^{*}p < 0.05$; $^{**}p < 0.01$; n.s., not significant). Quantitative protein abundance information (Mix-A vs. Glu) for the proteins highlighted in this figure is provided in Table S5.

Table S1. Purity analysis of Mix-A and Mix-B.

	OD_{260}/OD_{280}	OD_{260}/OD_{230}
Mix-A	2.09 ± 0.01	2.67 ± 0.01
Mix-B	2.21 ± 0.01	2.47 ± 0.01

For high purity: OD_{260}/OD_{280} = 1.9–2.2 and OD_{260}/OD_{230} > 2.0.

https://doi.org/10.3390/foods15020318

Table S2. Information on intracellular nuclease genes in *K. phaffii* X-33.

Subcellular location	Type of nuclease	Gene name	Function
Nucleus	Endonuclease	*RNASEH2A*	Endonuclease activity; participates in RNA–DNA hybrid ribonuclease activity and RNA metabolic processes.
		ERN1	RNA endonuclease and protein kinase activities.
		RNC	Ribonuclease III; cleaves double-stranded RNA and participates in rRNA metabolism.
		YSH1	Functions in mRNA metabolic processes, including the conversion of primary mRNA transcripts into mature mRNAs.
		FEN1	5′-flap endonuclease and 5′–3′ exonuclease activities; involved in DNA replication, DNA repair, and RNA primer removal.
	Exonuclease	*REX1*	Exonuclease activity; participates in rRNA processing.
		XRN2	5′–3′ RNA exonuclease activity; involved in DNA-templated transcription termination, mRNA processing, and rRNA processing.
Mitochondrion	Endonuclease	*RNZ*	3′-tRNA processing endoribonuclease activity.
		ENDOG	RNA and DNA endonuclease activities; participates in RNA catabolism and DNA recombination.
	Exonuclease	*ORN*	3′–5′ exoribonuclease activity; functions in mitochondrial genome maintenance.
		MSU1	Catalyzes the hydrolysis of phosphodiester bonds in RNA chains.
Cytoplasm	Endonuclease	*DOM34*	Endonuclease activity; participates in the nuclear-transcribed mRNA catabolic process.
	Exonuclease	*XRN1*	5′–3′ exoribonuclease activity; involved in the nuclear-transcribed mRNA catabolic process.
Vacuole	Endonuclease	*RNY1*	Ribonuclease T2 activity; catalyzes endonucleolytic cleavage of RNA to form nucleoside 3′-phosphates and 3′-oligonucleotides.
Exosome	Exonuclease	*RRP41*	3′–5′ exonuclease activity; participates in the nuclear-transcribed mRNA catabolic process and nonsense-mediated decay.
Cytoplasm	–	*GAPDH*	Reference gene for normalization.

Table S3. Primer sequences for RT-qPCR analysis of intracellular nuclease genes in *K. phaffii* X-33.

Gene name	Primers	Primer sequence (5′ to 3′)	Length/nt
RNASEH2A	Forward	GGCTGGAGTCGTGATGTCAGGT	22
	Reverse	GCTCCTCTTCGCCAAACACAACT	23
ERN1	Forward	CTCGGACAAGTGGGAAGCAGAAC	23
	Reverse	CCAGCAGCATTAGCCGTAGTAGC	23
RNC	Forward	GCCAAGTCCGCTCCCAAATACG	22
	Reverse	GCTCTAGTTCGGGCATCCTTAACAC	25
YSH1	Forward	AGTTGTGGTCGCATCTCCAGGTAT	24
	Reverse	TGTTGTGAAGGTATCTCGGTCGGT	24
FEN1	Forward	CGCAGAATTGGCTAAAGGAGGGAA	24
	Reverse	TCAGCAGTGGTCATGTGTCTCAAC	24
REX1	Forward	GTCTGAATCTTCGCAACCAGTCTCC	25
	Reverse	CCATCATAAGGCGGTGGTATCTGAAG	26
XRN2	Forward	CCAGAACACAAACCAGTCCCAGAG	24
	Reverse	GAGCAACACCATCAACGGCGAT	22
RNZ	Forward	GCGACCCTTGAGGATGAACTAATGG	25
	Reverse	GCTGCGAGAAATGCGTGAGAATC	23
ENDOG	Forward	GCTCATTATCTCCTCCAACAA	21
	Reverse	CAACTGATGGCGGACTTC	18
ORN	Forward	TCCAGCAACCGATAGTATG	19
	Reverse	CGTCAACCACCTCAAGAT	18
MSU1	Forward	TCTGGTGTCCGAGCTAATGATTGC	24
	Reverse	CTGCGACTGCCTGAACATCTCAA	23
DOM34	Forward	AGTGGAGTGGTGGTTCTTGTACCT	24
	Reverse	ACCTCATCGTCCTTGGCAATCAGA	24
XRN1	Forward	TGCTTACGGAACTCCTGCTACTGT	24
	Reverse	ATTGGTGTGTGCCTCCCTTTGTG	23
RNY1	Forward	TTGTCAGGTCAGTCAGGAT	19
	Reverse	TTGGTTCTTGCTCTTGTCA	19
RRP41	Forward	CCTCTGTAACAATCGGAGTCGTTGG	25
	Reverse	ACCTTAGCCAGTCTATCCTGTGGTAG	26
GAPDH	Forward	GGTAACATCATTCCATCTTCC	21
	Reverse	GACAACGGCATCTTCAGT	18

Table S4. Chemically synthesized RNA sequences used for the cultivation of *K. phaffii X-33*.

RNA	Primer sequence (5′ to 3′)	Length/nt
8 nt RNA	UGCCAGUA	8
12 nt RNA	AGCCAUGCAUGU	12
20 nt RNA	AUCUCGACCCUUUGGAAGAG	20
5′-Cy5-$RNA_{(20)}$	5′-Cy5-AUCUCGACCCUUUGGAAGAG	20

https://doi.org/10.3390/foods15020318

Supplementary Table

Table S5. Quantitative proteomic analysis of proteins involved in RNA oligonucleotide uptake and metabolism in yeast. This table provides normalized protein abundance values in yeast cells grown on Mix-A and glutamate (Glu), corresponding log_2 fold changes, and statistical significance. Separate sheets correspond to endocytosis, autophagy, RNA degradation, nucleotide metabolism, glycerophospholipid synthesis, and amino acid biosynthesis pathways. Proteins listed in this table correspond to those shown in Figures S6–S9.

https://doi.org/10.3390/foods15020318
